# Enhanced photocatalytic activity of Cu and Ni-doped ZnO nanostructures: A comparative study of methyl orange dye degradation in aqueous solution

**DOI:** 10.1016/j.heliyon.2023.e16506

**Published:** 2023-05-22

**Authors:** Md. Rashid Al-Mamun, Md. Zaveed Iqbal Rokon, Md. Abdur Rahim, Md. Ikram Hossain, Md. Shahinoor Islam, Md. Romzan Ali, Md Sadek Bacchu, Hiroki Waizumi, Tadahiro Komeda, Md Zaved Hossain Khan

**Affiliations:** a Department of Chemical Engineering, Jashore University of Science and Technology (JUST), Jashore 7408, Bangladesh; b Department of Civil and Environmental Engineering, University of Alberta, Edmonton T6G 1H9, Alberta, Canada; c Department of Chemical Engineering, Bangladesh University of Engineering and Technology (BUET), Dhaka 1000, Bangladesh; d Research Expert, Daffodil International University (DIU), Dhaka 1312, Bangladesh; e Department of Chemistry, Graduate School of Science, Tohoku University, Aramaki-Aza-Aoba, Aoba-Ku, Sendai 9808578, Japan; f Institute of Multidisciplinary Research for Advanced Materials (IMRAM, Tagen), Tohoku University, 2-1-1, Katahira, Aoba-Ku, Sendai 980-0877, Japan

**Keywords:** Photocatalysis, Methyl orange dye, Sol-gel method, Suspension reactor, UV light Illumination

## Abstract

Heterogeneous photocatalysis has been considered one of the most effective and efficient techniques to remove organic contaminants from wastewater. The present work was designed to examine the photocatalytic performance of metal (Cu and Ni) doped ZnO nanocomposites in methyl orange (MO) dye degradation under UV light illumination. The wurtzite hexagonal structure was observed for both undoped/doped ZnO and a crystalline size ranging between 8.84 ± 0.71 to 12.91 ± 0.84 nm by X-ray diffraction (XRD) analysis. The scanning electron microscope (SEM) and energy dispersive X-ray (EDX) revealed the irregular spherical shape with particle diameter (34.43 ± 6.03 to 26.43 ± 4.14 nm) and ensured the purity of the individual elemental composition respectively. The chemical bonds (O–H group) and binding energy (1021.8 eV) were identified by Fourier transform infrared (FTIR) and X-ray photoelectron spectroscopy (XPS) results respectively. The bandgap energy was decreased from 3.44 to 3.16 eV when Ni dopant was added to the ZnO lattice. The comparative photocatalytic activity was observed in undoped and doped nanocomposites and found to be 76.31%, 81.95%, 89.30%, and 83.39% for ZnO, Cu/ZnO, Ni/ZnO, and Cu/Ni/ZnO photocatalysts, respectively, for a particular dose (0.210 g) and dye concentration (10 mg L^−1^) after 180 min illumination of UV light. The photocatalytic performance was increased up to 94.40% with the increase of pH (12.0) whereas reduced (35.12%) with an increase in initial dye concentration (40 mg L^−1^) using Ni/ZnO nanocomposite. The Ni/ZnO nanocomposite showed excellent reusability and was found 81% after four consecutive cycles. The best-fitted reaction kinetics was followed by pseudo-first-order and found reaction rate constant (0.0117 min^−1^) using Ni/ZnO nanocomposite. The enhanced photodegradation efficiency was observed due to decreases in bandgap energy and the crystalline size of the photocatalyst. Therefore, Ni/ZnO nanocomposite could be used as an emerging photocatalyst to degrade bio-persistent organic dye compounds from textile wastewater.

## Highlights

• A facile sol-gel method was used to synthesize ZnO based photocatalysts.
• Photocatalysts were characterized by XPS, XRD, SEM with EDX, FTIR, and UV–Vis/DRS analyses.
• Hexagonal structure with crystalline size (17.21–8.84 nm) was observed by XRD analysis.
• Bandgap energy of photocatalysts were decreased in the range of 3.44 to 3.16 eV.
• Ni/ZnO exhibited maximum MO dye degradation (94.47%) under UV light illumination.
• Ni/ZnO showed excellent photostabilty and followed first order kinetics.

## Graphical abstract

**Figure:**
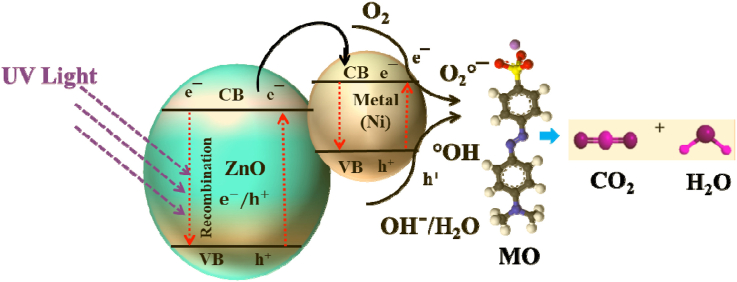

## 1 Introduction

Toxic liquid effluent discharge into the water bodies from textile industries causes a serious threat to human beings, livestock, and the aquatic ecosystem. Typically, the textile wastewater contains high suspended solids, high dissolved solids, heavy metals, organic dyes, solvents, microfibers, etc., which may be harmful, mutagenic, teratogenic, and carcinogenic [[1], [2], [3]]. Generally, the most widely used coloring compound in textile industries was the methyl orange (MO) dye which contains stable benzene rings, azo groups (–N=N–), and ethylated amino groups in their molecular structure. Due to hightly water-soluble in dye compounds, it was very difficult and expensive to remove these from wastewater using conventional treatment methods like chemical, physical, and biological ones [4,5]. Several drawbacks have been formulated in conventional processes like time-consuming, secondary sludge production, filtration requirement, higher chemical consumption, high energy consumption, incomplete conversion, low tolerance against toxic matters, high operating cost, and inability to remove organic dye pollutants [3,[6], [7], [8]]. Therefore, it was urgently required to remove certain types of dye compounds from effluents using an effective, economic, and eco-friendly method.

In this regard, advanced oxidation processes (AOPs) were known as a boon and green technology for the removal of organic dyes and phenolic compounds effectively without producing any harmful byproducts [[9], [10], [11], [12]]. The semiconductor materials were mostly used in the photocatalytic process for the removal of dye compounds in presence of solar or UV light illumination [13]. In the following mechanism, the photogenerated molecular reaction occurs on the catalyst surface during photocatalysis and generates holes and electrons in valence and conduction bands respectively. Then the conduction band electron reduced with pure O_2_ to produce superoxide anion radicals (O_2_°‾) whereas the valence band hole oxidized with H_2_O to form hydroxyls radical (°OH) respectively. These strong oxidative radicals (°OH, and O_2_°‾) were being utilized to degrade recalcitrant organic pollutants into less harmful products like carbon dioxide (CO_2_), water (H_2_O), and other minerals [5,7,14,15]. Recently, various metal oxides-based semiconductor materials like ZnO [16,17], V_2_O_5_ [18], CuO [19], TiO_2_ [20,21], WO_3_ [22], Fe_2_O_3_ [23], SnO_2_ [24], Al_2_O_3_ [25], ZrO_2_ [26], etc., have been employed in photocatalytic process. Though TiO_2_ was an effective catalyst for the removal of dye compounds in the presence of UV/Visible light irradiation. The major drawback of the TiO_2_ catalyst was the higher cost that limits its application on a commercial scale. Thus, it was extremely important to create an affordable catalyst for the removal of recalcitrant compounds that have increased adsorption, efficient degradation, and superior light harvesting properties [27]. Therefore, ZnO has been considered as a promising photocatalyst because of its low cost, abundance, chemical stability, affordability, non-toxicity, eco-friendly, long shelf-life, high electron mobility, and quantum yields properties [3,27,28]. Typically, it was an n-type semiconductor material belonging to the II-IV group that has a wide bandgap (∼3.4 eV) compared with TiO_2_ (∼3.20 eV) [29]. Furthermore, the ZnO can exist in three different phases namely hexagonal wurtzite, cubic sphalerite, and cubic rock salt. The hexagonal wurtzite phase was one of the most stable phases at ambient temperature and consisted of interpenetrating face-centered cubic (FCC) lattices of Zn and O [30]. Numerous approaches have been employed to synthesize ZnO nanoparticles (NPs) and sol-gel in addition to the heat treatment method was considered faster and simpler than other methods and has good control over stoichiometry at their molecular level [7,31,32]. Besides, the ZnO semiconductor has proven as the most superior photocatalyst that can be prepared in various shapes and sizes including nanorods, nanobowls, nanospheres, nanofilms, nanoparticles, nanodisks, nanotubes, nanoneedles, nanosheets, nanowires, nanowhiskers, etc. [[33], [34], [35]].

However, one of the biggest obstacles of ZnO NPs in photocatalysis was the large bandgap (∼3.40 eV) that inhibited the photocatalytic activity to the absorption of only the UV region. Furthermore, the visible light harvesting reponse of ZnO NPs is comparatively lower than that of TiO_2_ NPs [27,36]. To overcome these challenges, several strategies like coupling with semiconductors, surface modification, crystallinity improvement, sensitizing enrichment, and doping/co-doping with metals, and non-metals have been developed. The doping of transition metals (Cu, Ni, Co, Cr, Fe, Al) into ZnO lattice can lead to changes in their structural, morphological, optical, magnetic, and electrical properties [37,38]. Additionally, It promoted the absorption of visible light and prevented the rapid recombination of photogenerated electron-hole pairs by creating defective states in the bandgap [28,39]. In this case, the charge carriers were trapped in the defect sites and thus improved the interfacial charge transfer. The Cu and Ni metals have been used as effective modifiers for several visible light-responsive photocatalysts [36,40]. Besides, it reduced the bandgap energy of ZnO and changed the absorption edge, and consequently enhanced the photocatalytic performance of ZnO [38]. It can be seen that the ionic radius of Cu^2+^ (0.73 Å) and Ni^2+^ (0.69 Å) were almost similar that can easily incorporated into ZnO crystal as a deep acceptor in conjunction with neighboring oxygen vacancy. Therefore, it was commonly accepted that the doping of transition metal cations into the ZnO surface might be changed the coordination structure of Zn in the lattice [41]. According to the literature, the doping of Cu (3 wt%) in ZnO lattice improved the photocatalytic performance over the MO dye solution (20 mg L^−1^) and found photodegradation efficiency (39%) for a constant catalyst loading (1 mg mL^−1^) under UV light illumination [42]. Similar behavior was observed for the removal of crystal violet dye using Cu-doped ZnO nanocomposite. The authors claimed that the photodegradation efficiency increased with the increase in pH [43]. Another researcher reported the impact of Cu-doped SnO_2_ on the degradation of congo red under UV light illumination. The experimental result demonstrated higher degradation efficiency (91.32%) of using Cu-doped SnO_2_ than the bare SnO_2_ NPs [44]. [45] reported a more pronounced effect of Ag dopant on the photocatalytic activity than that of undoped ZnO NPs under UV light exposure [45]. The decreased bandgap up to 11.43% was observed by doping Cu and Ag metal into the ZnO lattice [46]. However, the impact of Cu dopant on photocatalytic degradation was rarely evaluated and to the best of our knowledge, very few studies were reported using metal-doped ZnO nanocomposites for assessing the photocatalytic performance of dye degradation. In this context, the study offered a preliminary roadmap for the efficient and long-term management of dye-containing wastewater.

Therefore, the primary objective of the current study was to assess the photocatalytic performance of metals (Cu or Ni) doped ZnO nanocomposites to methyl orange (MO) dye degradation under the illumination of UV light. To fulfill the above objective, the authors have used a facile sol-gel technique to synthesize ZnO, Cu/ZnO, Ni/ZnO, and Cu/Ni/ZnO nanocomposites. The crystal structure, morphology, chemical bonding, binding energy with chemical state, and optical properties of all nanocomposite samples were tested to realize the photocatalytic behavior of the nanocomposites. The comparative photocatalytic performance of nanocomposites was assessed under UV light illumination. The influence of various operating factors like catalyst dose, pH, and the initial dye concentration were evaluated. The reaction kinetics and the possible reaction mechanism based on dye degradation were also examined.

## 2 Materials and methods

### 2.1 Chemicals and reagents

The chemicals and reagents included zinc acetate dehydrate ((CH_3_COO)_2_Zn·2H_2_O) (Research lab, India, 98%), ethanol (C_2_H_5_OH) (Merck, Germany, 99.5%), copper (II) nitrate trihydrate (Cu(NO_3_)_2_·3H_2_O) (Merck, India, 99%), sodium hydroxide (NaOH) (Merck, Germany, 98%), nitric acid (HNO_3_) (Merck, Germany, 65%), nickel nitrate hexahydrate (Ni(NO_3_)_2_·6H_2_O) (Merck, Germany, 95%), and acetone (C_3_H_6_O) (Merck, India, 99.5%). All chemicals were analytical grade and required no further purification. Methyl orange (C_14_H_14_N_3_NaO_3_S) (USA, dye content 85%) was utilized as a model dye to assess the photodegradation activity and purchased from Sigma-Aldrich. Ultrapure water was used as a solvent for preparing the solution throughout every experiment.

### 2.2 Synthesis of undoped and doped ZnO nanocomposites

The glassware and apparatus were cleaned using ultrapure water, and acetone and dried (70°) in a micro-oven for 15 min. The zinc oxide (ZnO) was synthesized via sol-gel followed by the heat treatment technique. Generally, the synthesis process consisted of three steps including gel preparation, drying, and calcination. To synthesize metal-doped ZnO, the major precursors of all respective dopants (Cu or Ni) were mixed into the solution before the thermal treatment process. Initially, 8.78 g (0.2 M) of zinc acetate dehydrate was added with 100 mL of absolute ethanol and stirred continuously for 60 min in a beaker (250 mL) at ambient conditions [28,38]. In the following reactions, 100 mL of NaOH (0.2 M) was mixed with the solution and again stirred for 60 min to obtain zinc ions containing salt solution. Then the resulting white gel was kept in a closed chamber for 24 h followed by aging. Afterward, the white gel was allowed to centrifuge several times using ultrapure water at 5000 rpm for 15 min to remove residues. In the end, the resulting gel was held in an oven and dried for 120 min at each temperatures of 100 °C, and 110 °C for removing water and other volatile substances to the highest yield [47]. The solid white particle of ZnO was annealed at 400° for 1 h in a heating furnace (Thermo Scientific) and cooled naturally. The obtained sample was identified as being pure ZnO NPs. For the preparation of Cu/ZnO and Ni/ZnO nanocomposites, a similar procedure was adopted. In Cu/ZnO nanocomposite, initially, 20 mL of copper nitrate trihydrate (Cu(NO_3_)_2_·3H_2_O) (0.076 g) as precursor salt solution was prepared with ultrapure water and added drop-wise to the zinc acetate dehydrate, ethanol, and sodium hydroxide solution mixture with vigorous stirring (20 min) to maintain Cu (4 wt%) [13]. Similarly, for Ni/ZnO nanocomposite, 10 mL of nickel nitrate hexahydrate (Ni(NO_3_)_2_·6H_2_O) (0.058 g) solution was prepared with ultrapure water and added to the zinc acetate dehydrate, ethanol, and sodium hydroxide solution mixture with continuous stirring (20 min) to maintain Ni (2 wt%) [4]. For the preparation of Cu/Ni/ZnO nanocomposite, an equal volume of (Cu(NO_3_)_2_·3H_2_O) (0.076 g), and (Ni(NO_3_)_2_·6H_2_O) (0.058 g) was mixed drop-by-drop while continuous stirring to the aforementioned solution mixture [48]. Therefore, the remaining steps including drying and calcination was exactly similar to the ZnO NPs synthesis. The amount of metal dopant in the ZnO crystal structure was maintained relatively lower (4 wt% of Cu, and 2 wt% of Ni) for enhancing electrical, magnetic, and optical properties. The higher percentage of dopant can lead the potential changes in the properties through the formation of secondary phases and thus consequently reduces the effectiveness of doping [48]. The schematic diagram of synthesized ZnO-based nanocomposites is shown in Fig. S1 (Supplementary data).

### 2.3 Catalyst characterization

The structural studies were analyzed using XRD patterns (RINT2200, Rigaku, Japan) in the 2θ range of 20–80° with a scan rate of 0.02°/S at the surrounding temperature. The diffractograms were recorded with a Cu-Kα radiation tube at control configurations of wavelength (λ: 0.15406 nm), voltage (45 kV), and current (100 mA) respectively. Debye-Scherrer formula was applied to calculate the average crystalline size of all samples. The tetragonal formula was used to measure the lattice parameters based on miller indices. The study of binding energy and the new chemical state of all sample surfaces were performed by XPS (PerkinElmer, PHI 5600 XPS MA, USA), at a base pressure (4.0 × 10-8 Pa) in the analysis chamber. The excitation energy of Al Kα X-ray source (h**ν** = 1486.6 eV and 200 W) has been applied. Initially, the sample was positioned at an angle (θ = 45°) between the surface and the input lens of the analyzers. The calibration of the analyzer's energy has been tested with Au 4f7/2 core levels peak at 84.0 eV with a resolution of 0.125eV. The morphology and the individual elemental composition of samples were tested by SEM and EDX (ZEISS Gemini SEM 500, UK) analysis respectively. The sample specimens were prepared over aluminum stubs utilizing carbon tape with double adhesive. The micro and nano-level images were taken under controlled conditions (magnification: 130–200 kx; working distance: 6.6–6.9; and working voltage: 5.0 kV). The chemical bonds within the samples were determined by FTIR (NICOLET iS20, Germany) in the range of 4000 to 400 cm^−1^ using the KBr pellet technique. The absorption edge of the incident radiation was demonstrated in terms of wavelength. The optical absorption spectrum was measured using a double-beam spectrophotometer (Evolution-201, Thermo-Fisher-Scientific, UK) between 200 and 800 nm. Kubelka-Munk formula and Tau'c plots were used to calculate the bandgap energy which is demonstrated by plotting (F(R) X hv)^2^ vs. hv.

### 2.4 Photocatalytic experimentation

The photocatalytic performance was tested for the removal of MO dye under the illumination of UV light using photocatalysts like ZnO, Cu/ZnO, Ni/ZnO, and Cu/Ni/ZnO nanocomposites. A self-designed reactor consisted of four UV lights (80 W) with a parallel arrangement and was applied in a suspension photoreactor as an illumination source to ensure maximum light intensity. The photoreactor was made of a wooden box to prevent any stray radiation and the inside was covered with aluminum foil to improve reflection [41]. In the following step, the catalyst doses (0.060–0.210 g) were dissolved in 120 mL of dye-containing aqueous solution (10 mg L^−1^) of natural pH (8.7). To ensure the adsorption/desorption equilibrium of MO dye on the catalyst surface, the dye solution with the catalyst was stirred continuously for 20 min before UV light illumination. Afterward, the photocatalytic reaction was introduced at 180 min of UV light illumination. For additional photocatalytic studies, the effects of three factors catalyst dose, pH, and initial dye concentration were investigated. Nitric acid (HNO_3_), and sodium hydroxide (NaOH) were mixed into the solution to change the pH. A syringe was used to take off the solution (around 6 mL) from the reactor at predetermined intervals of 30, 60, 90, 120, 150, and 180 min. The specimens were filtrated using micro-filter paper (0.45 μm) and the change in concentration was analyzed by a spectrophotometer. The calibration curve (Fig. S2) of dye concentration was evaluated between the standard concentration of MO and recorded UV absorbance at the wavelength of 464 nm. The experimental setup of the photocatalytic reactor with labeling is shown in Fig. S3.

The photocatalytic efficiency of MO dye was calculated as stated in Eq. (1), where C_o_ and C_t_ (mg L^−1^) indicate the initial and final concentration of MO dye after photoreaction, respectively.

(1) $$Dye\;degradation\;efficiency,\left(\%\right)=\left(C_{o}-C_{t}\right)/C_{o}\times 100$$

## 3 Results and discussion

### 3.1 Photocatalysts characterization

**Fig. 1:**
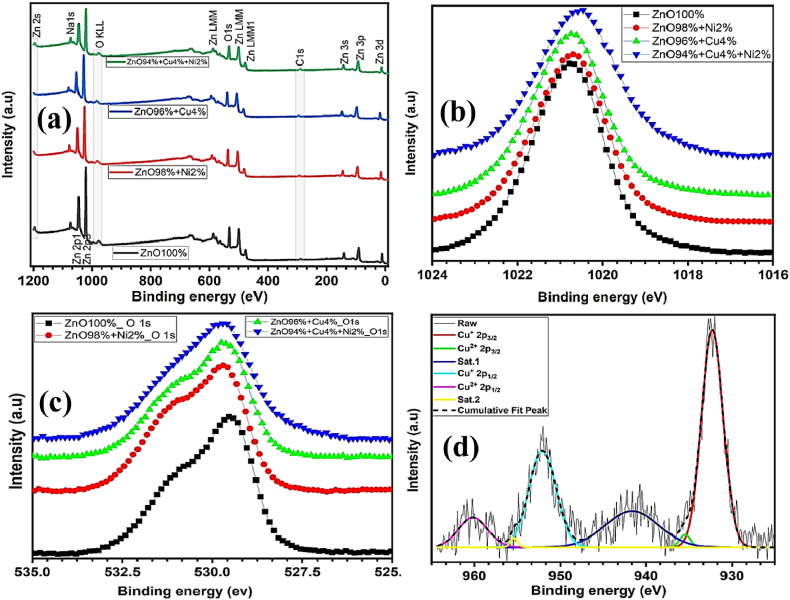
XPS spectra of pure ZnO nanoparticles and peaks of different doped peaks for Ni, Cu. (a) Survey XPS spectra of pure ZnO nanocomposite and different doped nanocomposites of Ni and Cu (b) XPS spectra Zn 2p_3/2_, (c) O1s showing shift in binding energy following different doping, and (d) deconvoluted spectra of Cu 2p at ZnO_96%_/Cu_4%_.

The incorporation of Ni or Cu into the ZnO nanocomposites has been explained by the formation of chemical bonds and the electronic structure. The samples valence-band and core-level XPS spectra is shown in Fig. 1. As shown in Fig. 1, the binding energies of Zn 2p_3/2_, and O 1s were found to be 1021.8 and ≈ 529.5 eV, respectively, based on intensive lines of all samples. Comparing the binding energies of these lines indicated similar XPS core levels of matrix elements of the ZnO wurtzite (hexagonal) structure [49]. A very weak peak was positioned at ≈853.8 eV which corresponds to the Ni 2p states in the Ni_2%_/ZnO_98%_, Cu_4%_/Ni_2%_/ZnO_94%_, while the peak at ≈932.6 eV in the spectrum corresponds to Cu 2p of sample Cu_4%_/ZnO_96%_, and Cu_4%_/Ni_2%_/ZnO_94%_ respectively, (Fig. 1a). Additionally, the intensity of Ni or Cu metal was also observed very weak due to the lower amount of doping concentration. The individual scan of Ni metal was confirmed and the deconvoluted peaks were consistent with XRD studies. Fig. 1b depicted the comparative study of XPS spectra for the composition and chemical bond configuration of different percentages of Ni or Cu. The high-resolution spectrum (Fig. 1b) indicated the binding energy of Zn 2p_3/2_ for Zn^2+^ states in ZnO around 1021.6 eV which was closely matching with the standard data of ZnO. The binding energy was slightly shifted to the lower binding energy due to the incorporation of Ni (2%) and Cu (4%) into the ZnO structure. Based on the metal reactivity series, the Zn was most reactive compared to Ni and Cu metals. There was no probability of replacing Zn with Ni or Cu rather than Zn, and therefore, the O1s showed a little peak shift towards higher binding energy (Fig. 1c). As shown in Fig. 1d, the Ni and Cu peak shifted towards the lower binding energy and oxidation state than compared to the pure compounds of these metals. So there was a possibility of sharing the oxygen of ZnO with both Ni and Cu. From, XPS results, the authors considered a partial charge distribution that occurred due to the presence of Cu and Ni in ZnO. The deconvoluted XPS spectra for Ni and Cu 2p in (a) ZnO_98%_/Ni_2%_, (b) ZnO_94%_/Cu_4%_/Ni_2%_, and (c) ZnO_96%_/Cu_4%_ are shown in Fig. S4.

**Fig. 2:**
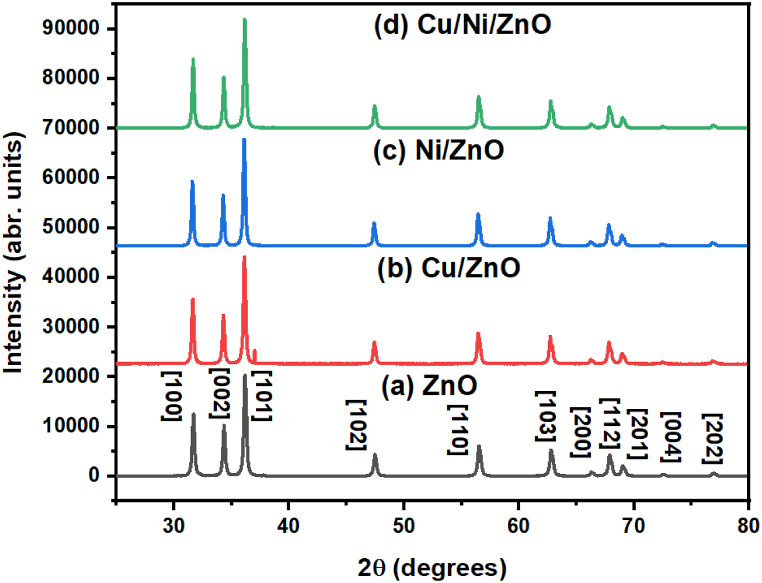
XRD spectra of synthesized photocatalysts; (a) ZnO, (b) Cu/ZnO, (c) Ni/ZnO, and (d) Cu/Ni/ZnO.

Fig. 2 depicted the XRD patterns of all samples to determine the crystal phase, crystallinity, and phase purity. As shown in Fig. 2a, the 2θ peaks of 31.70°, 34.38°, 36.16°, 47.46°, 56.52°, 62.81°, 66.35°, 67.91°, 69.11°, 72.64°, and 76.96° which correspond to {100}, {002}, {101}, {102}, {110}, {103}, {200}, {112}, {201}, {004}, and {202} (JCPDS file card 36–1451) diffraction planes of hexagonal wurtzite phase of ZnO [28]. Almost similar trends of XRD patterns were found due to the addition of metal (Cu or Ni) into ZnO photocatalysts (Fig. 2b–d). No significant diffraction peaks of any other were observed that indicate higher phase purity of nanocomposites. Additionally, these samples preserved all Bragg reflection planes due to the improvement of ZnO with Cu and Ni metals that indicate the integral of crystal structure. This was observed because of its almost close ionic radius of Cu^2+^ (0.73 Å), and Ni^2+^ (0.69 Å) to Zn^2+^ (0.74 Å) which determined the easy incorporation of metal into the ZnO crystal lattice [30]. However, the XRD result revealed the coexistence of diffraction planes that ensured the successful compositing of metal dopant into the ZnO crystal surface with no effect on the overall crystal structure [38]. The Eq. (2) represented the Debye Scherrer formula, where the wavelength of X-ray radiation was λ, the diffraction angle of Bragg was θ, the width at half maximum (FWHM) peak was β, and the Scherer constant was K (0.89), respectively. The crystalline size (average) was calculated and found to be 12.91 ± 0.84, 9.24 ± 0.46, 8.84 ± 0.71, and 9.54 ± 0.67 nm for ZnO, Cu/ZnO, Ni/ZnO, and Cu/Ni/ZnO nanocomposites, respectively (Table 1). However, the crystallite sizes of photocatalysts decreased slightly with the addition of metal dopants into the ZnO structure. The possible reason might be the incorporation of foreign impurities of Cu^2+^, and Ni^2+^ in the ZnO lattice that reduced the subsequent growth rate and nucleation of ZnO [50,51]. The crystal lattice parameters (a and c) were calculated by using Eq. (3), where miller indices were h, k, and l, and lattice spacing of inter-planar distance was d. The values of the measured lattice parameter were discovered to be a = 3.2534 Å and c = 5.6351 Å for the pure ZnO NPs and a = 3.2460 Å and c = 5.6224 Å for the Cu/Ni/ZnO nanocomposite. As we can seen the lattice parameters (a and c), unit cell volume, and dislocation densities were slightly decreased in metal dopant nanocomposites than that of the pure ZnO lattice structure. This was formed due to the substitutional incorporation of Cu^2+^ and Ni^2+^ ions with lower ionic radius into the Zn^2+^ site [50]. By comparing the obtained values with the standard lattice volume, we can suggested that the volume of dopant-assisted nanocomposites was almost closer to that of pure ZnO. Thus, it indicated the most stable lattice [39]. The unit cell volume was calculated using V = 0.866a^2^c and found to be the decreased order with the addition of metal dopant into the ZnO lattice. This can be explained by the shift of peak to the lower theta values and increased in the inter-planar distance (d-spacing) [52]. The dislocation densities were measured by using 1/d^2^ and are summarized in Table 1.

(2) $$D=\frac{K\lambda }{\beta \;\cos\;\theta }$$

(3) $$\frac{1}{d^{2}}=\frac{4\left(h^{2}+hk+k^{2}\right)}{{3a}^{2}}+\frac{l^{2}}{c^{2}}$$

**Table 1:** An overview of the theoretical-physicochemical characteristics of synthesized ZnO based photocatalysts.

Samples	Method	Structure phase	Crystalline size (nm)	Diameter (nm)	Dislocation density (nm)^−2^	Lacttice spacing, d_hkl_ (Å)	Lattice parameters (Å)		Volume of unit cell, (Å)^3^	EDX mapping				Bandgap (eV)
							a	c		Zn (mass%)	O (mass %)	Cu (mass %)	Ni (mass %)	
ZnO	Sol-gel	Hexagonal	12.91 ± 0.84	26.43 ± 4.14	0.0059	2.8176	3.2534	5.6351	45.9146	82.88	17.12	–	–	3.44
Cu/ZnO	Sol-gel	Hexagonal	9.24 ± 0.46	33.46 ± 5.57	0.0117	2.8024	3.2359	5.6047	45.1776	82.45	16.41	1.14	–	3.30
Ni/ZnO	Sol-gel	Hexagonal	8.84 ± 0.71	34.43 ± 6.03	0.0127	2.8109	3.2412	5.6217	45.0757	72.09	27.71	–	0.20	3.16
Cu/Ni/ZnO	Sol-gel	Hexagonal	9.54 ± 0.67	30.99 ± 4.58	0.0109	2.8112	3.2460	5.6224	45.6020	81.45	15.98	1.97	0.60	3.35

**Fig. 3:**
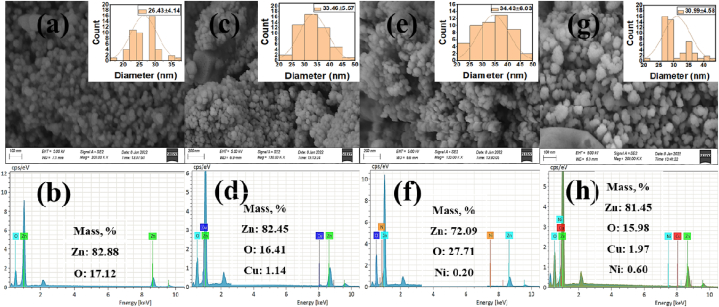
SEM image with particle size diameter of synthesized photocatalysts; (a) ZnO, (c) Cu/ZnO, (e) Ni/ZnO, and (g) Cu/Ni/ZnO; and EDX analysis (b) ZnO, (d) Cu/ZnO, (f) Ni/ZnO, and (h) Cu/Ni/ZnO.

The surface morphologies of all ZnO-based samples were performed using SEM analysis as depicted in Fig. 3. As shown in Fig. 3a, a relatively smooth surface, porous bulk structure, and perfectly spherical shape nanoparticles with uniformly distributed over the entire surface were observed. The morphology of metal (Cu or Ni) doped ZnO nanocomposites was comparatively distinct from that of undoped ZnO NPs (Fig. 3(c, e, and g)). It can be seen that higher smoothness, rigidness, highly aggregated, and more porous surface area was found in dopant-assisted ZnO nanocomposites. The well-dispersed aggregated can be beneficial in photocatalysis improved the adsorption of dye molecules on the catalyst surface and supported the entrance of light in the photodegradation process [4]. Similar results were observed in the literature [38]. Based on the SEM image, the average diameter was determined to be 22–30, 27–38, 28–40, and 26–36 nm for ZnO, Cu/ZnO, Ni/ZnO, and Cu/Ni/ZnO nanocomposites, respectively. The collected particles' crystallinity and diameter were discovered to be found comparatively consistent with XRD results (Table 1). It was reported that such kind of heterojunction enhanced active sites by trapping charge carriers, inhibiting electron-hole recombinant rate, and consequently improved the photodegradation efficiency of dye [28]. The individual elemental compositions of all samples were analyzed using EDX spectra. Fig. 3(b, d, f, and h) depicted the EDX spectra of all synthesized samples. The EDX spectra ensured the presence of identified elements such as Zn, O, Cu, and Ni that represented homogeneous distribution into the ZnO surface. The mass percentages of all synthesized samples are provided in Table 1. It was observed from the EDX pattern that the percentage composition by mass of Zn, O, Cu, and Ni in the as-synthesized sample was 81.45%, 15.98%, 1.97%, and 0.60%, respectively. It was found that the % of Zn and O elements decreased with the addition of metal dopant onto the ZnO surface. Additionally, the mass percentages obtained by EDX analysis were in rational agreement with the expected values. Furthermore, these spectra confirmed the purity of all nanocomposites.

**Fig. 4:**
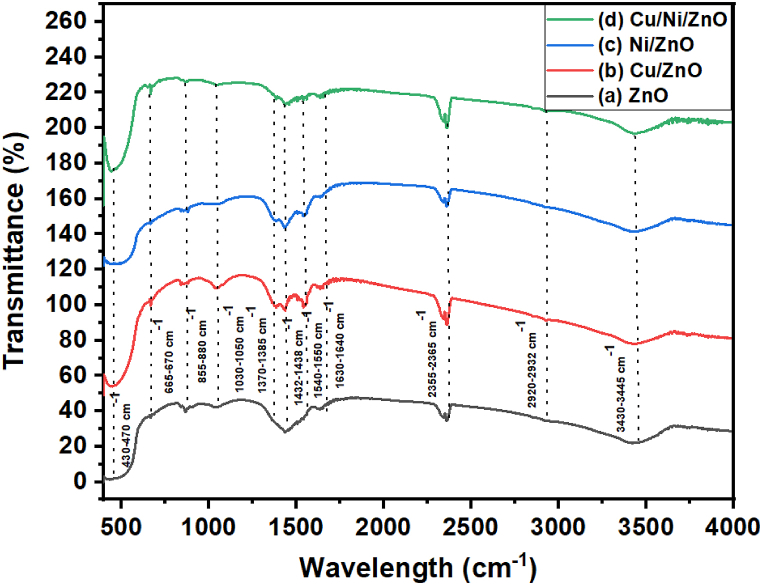
FT-IR spectra of synthesized photocatalysts; (a) ZnO, (b) Cu/ZnO, (c) Ni/ZnO, and (d) Cu/Ni/ZnO.

Fig. 4 indicated the FTIR spectrum of ZnO, Cu/ZnO, Ni/ZnO, and Cu/Ni/ZnO samples to show different chemical bonds. The strong peak at 3430-3445 cm^−1^ represented the stretching and bending vibration of O–H groups with adsorbed water molecules. The small and weak absorption peaks at 2920-2930 cm^−1^ were assigned to the vibration and symmetric modes of the Ni–O–C and C–H bond [4,34]. The peaks observed at around 2356-2365 cm^−1^ were ascribed to the adsorbed CO_2_ on the surface of the photocatalysts. The absorption peaks at 1630-1640 cm^−1^, 1540-1550 cm^−1^, 1432-1438 cm^−1^ were assigned to the COO-metal bond, C–OH vibrations (twisting, wagging), and C–O stretching, respectively [4]. The peak at around 1030-1050 cm^−1^ can be ascribed to the stretching vibrations of H–O–H molecules, and normal polymeric O–H bonds in the Zn–O or Cu/Ni–Zn–O lattice. The band located at 800 cm^−1^ was associated with C–C stretching vibration [50]. The observed peak in the range of 430–670 cm^−1^ equivalented to the stretching vibration of metal-oxygen that confirmed the formation of Zn–O, Ni–ZnO, and Cu–ZnO modes. Hence, the authors believed that metal ions have successfully incorporated into the crystal lattice of ZnO. This bond formation was observed due to the interaction between the free electrons on the surface of ZnO NPs with some unpaired electrons of metal dopants [38]. The amount of O–H bonds in photocatalysts' surfaces plays a key role by providing an electron to the valence band and thus formed a highly reactive hydroxyl radical (°OH) that leads to the mineralization of organic dye compounds [53].

To evaluate the optical characteristics of all samples, the absorbance-diffuse reflectance spectra, and bandgap energy measurements were tested at surrounding temperatures and the results are displayed in Fig. S5. The absorption edge was observed in the range of wavelengths (350–400 nm) that indicated the intrinsic band transition [28]. The more significant red shift in the absorption in the UV region with respect to the inter-band transition of ZnO NPs was found in dopant-assisted nanocomposites that confirmed the generation of larger charge carriers (Fig. S5a). Besides, the broad absorption was seen due to interfacial charge transfer and d–d transition of metal ions [50]. The Ni/ZnO nanocomposite showed the maximum broad shift in the absorption spectrum towards the visible region [28]. In reflectance spectra, it was observed that the reflectance percentage was greatly increased with the incorporation of Cu or Ni metal into the ZnO crystal lattice which revealed greater intensity of absorption at the corresponding edge. It can be seen that the % of reflectance in the visible region was increased with the addition of metal dopant and was found to be 32%, 50%, 57%, and 55% for ZnO, Cu/ZnO, Ni/ZnO, and Cu/Ni/ZnO nanocomposites, respectively. This finding was observed due to losses of absorption and lower scattering of nanocomposites [39]. It was reported that the optical absorption edge and absorption intensity of the photocatalyst significantly influenced photocatalytic activity [52]. The bandgap energy (E_g_) of all samples was measured from the absorption spectra using a Kubelka–Munk function and Tau'c plot that provided a relation between the absorbance coefficient (α) of the catalyst and the incident photon energy (hv) [54]. The results were obtained by Eq. (4), where bandgap energy E_g_, constant was K, frequency of the incident radiation was v, and Plank's constant was h. The value of n generally depended on the nature of transition and used n = 1/2, and 2.0 for direct and indirect transition, respectively. The estimated E_g_ of all nanocomposites were found to be 3.44 eV, 3.30 eV, 3.16 eV, and 3.35 eV for ZnO, Cu/ZnO, Ni/ZnO, and Cu/Ni/ZnO nanocomposites, respectively. The E_g_ of all samples were determined using Tauc plot is shown in Fig. S6. Based on the literature search, the E_g_ of NiO was approximately 3.8 eV, while the band gap of CuO was around 1.23 eV [55]. This was significantly red-shifted from the bulk ZnO NPs (3.44 eV) which can be elucidated in the quantum size effect and small confinement of electrons in the energy levels [2,56]. The lowest E_g_ was observed in Ni/ZnO nanocomposite and it can be explained based on ionic radius. The ionic radius of Zn^2+^ (0.74 Å) and Ni^2+^ (0.69 Å) revealed the wide range of solubility of Ni into ZnO NPs [56]. Therefore, it suggested that Ni^2+^ ion worked as defect sites in the valence band to decreased the band gap. Furthermore, it was observed due to the formation of sp–d exchange interactivity and electronic transition level with a metal dopant to the host's conduction band (ZnO) photocatalyst [51]. Due to the lower electronegativity of Ni dopant than Zn, it leads to the creation of Ni impurity levels within the ZnO structure [55]. The Ni impurity levels act as intermediate energy levels that can facilitate the transition of electrons from the valence band to the conduction band, effectively reducing the E_g_ of the material [57]. It was reported that the lower E_g_-containing nanocomposite exhibited favorable visible light absorption, and thus enhanced photocatalytic performance [53].

(4) $$\alpha hv=K{\left(hv-E_{g}\right)}^{n}$$

### 3.2 Photocatalytic studies

The photocatalytic performance was studied for the removal of MO dye in aqueous suspension using ZnO, Cu/ZnO, Ni/ZnO, and Cu/Ni/ZnO nanocomposites in presence of 180 min illumination of UV light. The nanocomposites were initially placed in the reactor at the range of 0.060 g–0.210 g with a constant dye solution (10 mg L^−1^) at natural pH (8.7). The photoreactivity was influenced by several operating parameters like dosage of photocatalyst, pH of the solution, and the initial dye concentration was also evaluated.

#### 3.2.1 Impact of different photocatalysts

**Fig. 5:**
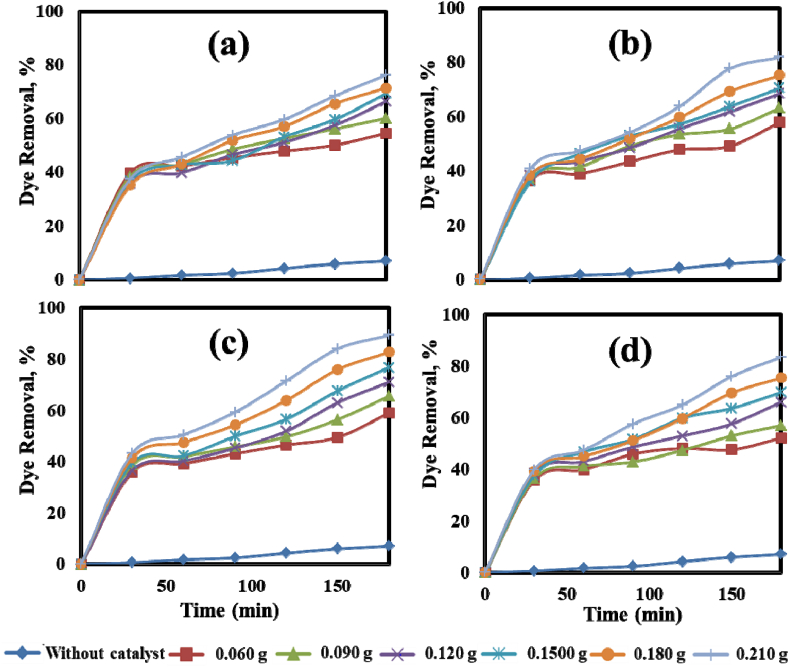
Photocatalytic performance of MO dye under the illumination of UV light; (a) ZnO, (b) Cu/ZnO, (c) Ni/ZnO, and (d) Cu/Ni/ZnO (Experimental condition: dose of photocatalyst: 0.060–0.210 g, pH: 8.7, initial MO dye conc.: 10 mg L^−1^).

The comparative photocatalytic performance of ZnO, Cu/ZnO, Ni/ZnO, and Cu/Ni/ZnO photocatalysts for the removal of MO dye in an aqueous suspension was studied under 180 min illumination of UV light and the outcomes are displayed in Fig. 5. Generally, the experiments were carried out both in UV light and dark conditions. It can be seen that no significant dye degradation (less than 8%) was noted without the use of a photocatalyst after 180 min illumination of UV light. Additionally, the photocatalysts adsorbed a small amount of dye (11%) without the light source before the illumination of the light. It was evident that photocatalysts plays a significant role in the photocatalytic process. The photodegradation efficiency of MO dye was 76.31%, 81.95%, 89.30%, and 83.39% for ZnO, Cu/ZnO, Ni/ZnO, and Cu/Ni/ZnO photocatalysts, respectively, for a particular dose (0.210 g) at pH (8.7) after 180 min illumination of UV light (Fig. a–d). It can be found that the photodegradation efficiency was increased with the increase in the concentration of photocatalysts (0.060 g–0.210 g) and then decreases in the suspension reactor. Furthermore, it was evident that a Ni-doped ZnO photocatalyst degraded MO dye at a significantly faster rate than pure ZnO NPs [58]. The lowest and the maximum photodegradation efficiency was found in undoped ZnO NPs, and 2 wt% Ni-doped ZnO nanocomposite, respectively that indicated the photogenerated electron-hole charge carriers were strongly inhibited by using nanocomposites [53]. The order of the photocatalytic degradation of dye was ZnO < Cu/ZnO < Ni/ZnO < Cu/Ni/ZnO which revealed the order of decreased particle size with bandgap energy. The obtained lower crystal sizes were measured from XRD results (Table 1) also enhanced the photocatalytic activity of the photocatalysts. The formation of functional groups like Zn–O–Ni and Zn–O–Cu bonds on the ZnO surface enhanced the photocatalytic performance [58]. The lower bandgap allowed the photocatalyst to enhance the photogenerated charge carriers and light absorption [53]. Besides, the transition metal (Cu and Ni) doped ZnO nanocomposite has generated more oxygen vacancy that lead to higher electron-hole recombination and thus consequently enhanced the effectiveness of photocatalytic dye degradation [59]. Furthermore, it was observed that the color of MO dye changed from orangish-yellow to colorless with the increase of the photocatalyst in the suspension reactor. These findings might be seen due to the available active sites and effective absorption of light that generated abundant oxidative radicals to increase MO dye degradation [56]. Based on the literature, the doping of metal in ZnO enriched the valence band (VB) significantly in terms of the Fermi level that may lead to increased electron transport and higher photocatalytic degradation [58]. Therefore, a series of highly reactive species like hydroxyl radical (°OH), superior electrostatic interactions, hydrogen peroxide, and superoxide anion radicals (O_2_^°‾^) were involved in an organic MO dye pollutant degradation [54].

### 3.3 Impact of pH

**Fig. 6:**
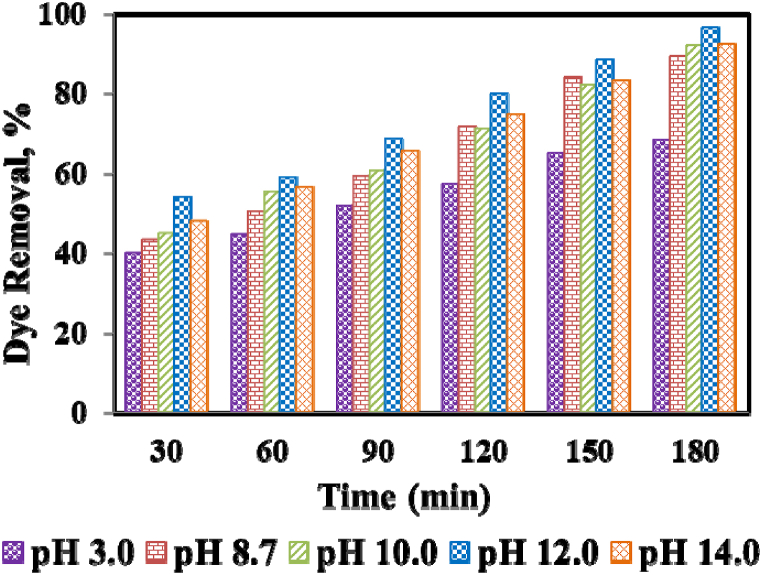
Impact of pH on MO dye removal (Experimental condition: 0.210 g of Ni/ZnO photocatalyst, dye concentration: 10 mg L^−1^, pH: 3.0, 8.7, 10.0, 12.0 and 14.0).

Among various operating conditions, the pH of the dye solution plays a key factor in evaluating the photodegradation reaction in an aqueous medium. To explore the impacts of pH on the photocatalytic process, tests were carried out at pH 3.0, 8.7, 10.0, 12.0, and 14.0, and all experimental conditions were kept fixed (catalyst dose: 0.210 g of Ni/ZnO nanocomposite, dye concentration: 10 mg L^−1^, and UV light illumination: 180 min). Initially, the impact of high and low pH was evaluated in absence of a photocatalyst and there were no significant changes in MO dye concentration observed after 180 min illumination of UV light. The obtained results of pH on MO dye degradation using Ni/ZnO nanocomposite are shown in Fig. 6. It can be seen that with the increase of pH from 8.0 to 12.0, the photocatalytic activity of dye was increased followed by acidic and neutral pH. The photodegradation efficiencies were observed around 89.30%, 91.99%, and 94.40% for pH 8.7, 10.0, and 12.0, respectively. The pronounced influence of pH on MO dye degradation was obtained with the order of pH 3.0 < 8.7<10.0<12.0>14.0. The experimental results exhibited maximum degradation efficiency in the alkaline medium followed by the neutral and acidic medium. This observation was found due to the availability of more OH‾ ions in the solution that generated higher °OH radicals by coupling with positive holes of the semiconductor [60]. The rate of °OH radical formation was decreased at pH 14.0 due to Coulomb repulsion between the OH‾ and the negatively charged surface of the photocatalyst and thus leading to lower photodegradation efficiency [56].

The surface charge property of a photocatalyst plays an important role in photocatalytic reactions which directly affects its photoactivity. The photocatalysts surface charge was zero at this point was known as point zero charge (pH_PZC_) [36]. It can be seen that the pH_PZC_ of ZnO NPs was 9.0 [61]. At pH > pH_PZC_, the catalyst surface was positively charged and a protonation reaction occurred while a deprotonation reaction occurred at pH < pH_PZC_ that made the surface negatively charged [36]. Methyl orange was an anionic dye, and the positively charged particles improved the transfer of photogenerated electrons that might react with an adsorbed O_2_ molecule to form superoxide anion radicals (O_2_°‾). Similarly, this process could also suppressed the photogenerated electron-hole recombination and produced more hydroxyl radical (°OH) through the reaction with water. Therefore, these charge carriers could be enhanced photocatalytic performance. In such alkaline conditions, the adsorption of MO dye became stronger due to the attractive coulombic between the photocatalyst surface and dye solution [38]. Excess of OH‾/OH^+^ ions can suppress the chain reaction of radicals’ species, and thus indicated lower photocatalytic performance.

### 3.4 Impact of dye concentration

**Fig. 7:**
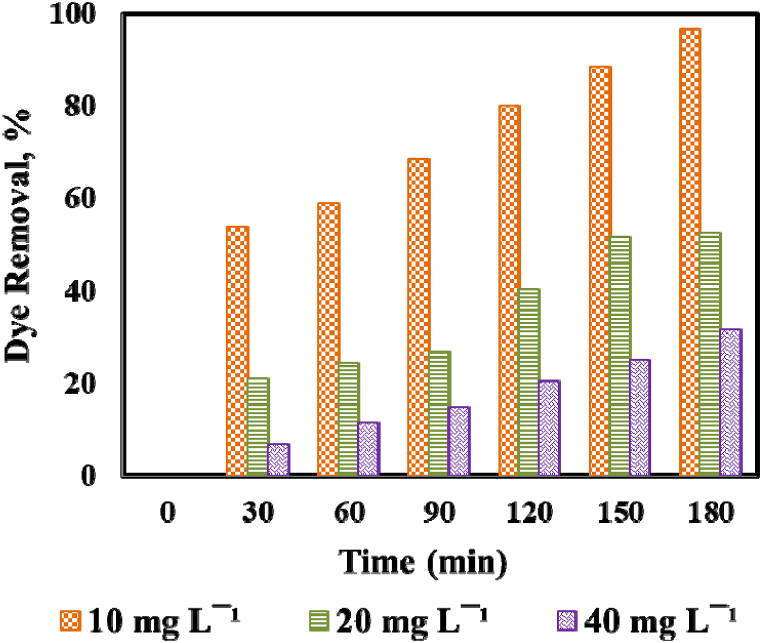
Impact of initial dye concentration on MO dye removal; (Experimental condition: 0.210 g of Ni/ZnO photocatalyst, dye conc.: 10–40 mg L^−1^, pH: 12.0).

The photocatalytic performance of dye was strongly influenced by initial dye concentration. More than 90% of dye degradation was obtained using Ni/ZnO nanocomposite while keeping all other operating conditions (dye concentration: 10 mg L^−1^, pH: 12.0, catalyst dose: 0.210 g, and the illumination of UV light: 180 min). Therefore, the photocatalytic tests were evaluated at higher initial dye concentrations like 20 and 40 mg L^−1^ and the resulting results are displayed in Fig. 7. The efficiency of dye photodegradation was determined to be 94.47%, 63.34%, and 35.12% for 10, 20, and 40 mg L^−1^, respectively, after 180 min of UV light illumination indicated the removal performance was decreased with the addition of initial dye concentration in suspension reactor. This was observed due to the adsorption on the available active sites of the catalyst surface by MO dye molecules and the solution turned more concentrated colored and therefore reduced the formation of °OH radicals [56]. However, the competitive adsorption of OH‾ and O_2_ on the same sites was decreased leading to the lower formation of °OH and O_2_°‾ radicals and consequently reduced the photodegradation rate [62]. Furthermore, the length of photon penetration was reduced with the addition of initial dye concentration consequently fewer photons will reach the catalyst surface to trap the pollutants and thus leading to lower photodegradation efficiency [28,56].

### 3.5 Reusability and stability of photocatalyst

The stability of the photocatalyst was a key factor for the application of a structural photocatalytic reactor. To study the reusability and stability of Ni/ZnO photocatalyst, four consecutive cycles were tested with respect to the above-mentioned conditions. In the following step, the recollected catalyst was filtrated and subsequently redistributed in the MO dye-containing solution for the subsequent UV light illumination cycle. It was found that the photodegradation efficiency was not reduced significantly after four successive cycles. The obtaining photodegradation efficiency was to be 94.4%, 92.71%, 86.36%, and 81.29% for 1st, 2nd, 3rd, and 4th cycles, respectively, revealed the photostability of Ni/ZnO photocatalyst. This was developed as a result of the intermediate products adsorption to the catalysts active sites, and the weight loss of the catalyst [17]. This outcome suggested that the Ni/ZnO photocatalyst was recyclable and has photostability for the photocatalytic MO degradation process.

## 3.6 Comparison and validation of dye degradation

The comparison of photocatalytic performance of undoped ZnO, Cu/ZnO, Ni/ZnO, and Cu/Ni/ZnO nanocomposites are shown in Fig. S7. As shown in Fig. S7, the Ni/ZnO nanocomposite demonstrated highest photocatalytic performance than other photocatalysts due to the large concentration and well dispersity of Ni metal into the ZnO surface [38]. The possible reason for the highest activity might be the incorporation of metallic Ni particles into the ZnO lattice and distributed onto the ZnO surface. FESEM images exhibited higher irregular, dispersity, and porous morphology of Ni/ZnO photocatalysts than that of other photocatalysts. It was reported that the good dispersity of metal (Ni) on the ZnO photocatalyst surface showed higher photocatalytic activity [63]. It was observed that the particle size was greatly decreased with the addition of metal dopant into the ZnO lattice and the lowest crystal/particle size was observed in Ni/ZnO photocatalyst. It was seen that the smaller particle size and the shifting of their absorbance to the visible region resulted in an enhancement of their photodegradation efficiency [64]. There was no shift in the Zn–O bond after Ni doping and the presence of more °OH radicals was the key factor for higher photocatalytic performance. The absorption spectrum shifted towards the longer wavelength due to their crystal structure and quantum confinement effect that indicated strong light absorption capacity. XPS analysis determined the binding energy 1021.6 eV for Zn 2p_3/2_ of Zn^2+^ states in ZnO which was closely monitored with the standard data of ZnO. As we incorporated Ni (2%) and Cu (4%), the binding energy of ZnO slightly shifted to the lower binding energy.

A comparison of our experimental findings with other published works in the literature is shown in Table 2. Vaiano et al. [51] reported the Cu-doped ZnO photocatalyst prepared by the chemical-precipitation method for the photodegradation of As(III) to As(V) under UV light illumination. A 1.08 mol% doping of Cu decreased the average crystallite size (33-32 nm) and bandgap energy (3.2–2.92 eV) and exhibited a low photodegradation efficiency (52.0%) even after 120 min of light exposure [51]. As shown in Table 2 [4], synthesized Ni-doped ZnO by using the sol-gel method for the removal of acid blue 1 (AB1) dye under UV light. The authors asserted that the spherical structure of ZnO NPs almost changed to homogenous hexagonal NPs with the addition of Ni dopant. The impacts of operating conditions like catalyst dose, pH, and initial dye concentration on dye were studied based on dye degradation. They reported the highest dye degradation by using Ni/ZnO photocatalyst [4]. The Cu-doped ZnO nanorod was successfully prepared with a chemical growth method for the treatment of MB dye under the illumination of UV light. A slight change in crystal lattice was observed after Cu doping, as well as bandgap energy, decreased up to 10.4%. The photodegradation efficiency was found to be 60.0% after 180 min illumination of UV light [65]. Uma et al. [56] investigated the Ni-doped ZnO NPs by electrically synthesized route for the application of indigo carmine dye degradation. The average crystalline size of Ni/ZnO NPs was 78.3 nm and exhibited the highest absorption at 340 nm. The authors claimed the efficiency of indigo dye degradation was found to be 84.8% after 90 min illumination of UV light [56]. As shown in Table 2, earlier reported works supported our results of obtaining the highest removal of dye using Ni/ZnO photocatalyst. However, the removal was higher than the reported works which might be the reason for using different uses ratios of Ni during synthesis. Therefore, it can be concluded that the Ni/ZnO photocatalyst might be superior compared to other photocatalysts for the treatment of organic dye dye-containing wastewater from textile effluents.

**Table 2:** Performance of the current works's photodegradation in comparison to other published studies.

Dye compound	Photo catalyst	Photocatalyst dose	Synthesis technique	Synthesis reagents and reaction conditions	Doping conc.	Catalyst surface area, (m^2^/g)	Modified bandgap, and λ value	Particle size, nm	Light source	Experimental conditions	Illumination time, min	Photodegradation efficiency, %	Ref.
Methylene blue	Cu/ZnO	3 g/L	Precipitation method	Cu(CH_3_COO), ZnC_4_H_6_O_4_, calcined at 600 °C for 2 h.	1.08 mol%	6.6	2.92 eV, (300–900 nm)	32–33	10 W Visible LED strip	pH: 7.0, dye vol.: 100 mL, dye conc.,: 10.0 mg L^−1^	120	52.0	[51]
Acid Blue 1	Ni/ZnO	1.07 g/L	Sol-gel method	(Ni (NO_3_)_2_⋅6H_2_O), (Zn (CH_3_COO)_2_⋅2H_2_O), the gel was dried at 80 °C for 18 h.	1.12 wt%	N/A	2.97 eV, (368 nm)	19.19–24.79	6 W Hg lamp	pH: 7.77, dye vol.: 100 mL, dye conc.,: 10.0 mg L^−1^	180	90.23	[4]
Methylene blue	Cu/ZnO	5–20 g/L	Chemical growth method	(Cu (OAc))_2_, (ZnC_4_H_6_O_4_), the solution was kept in oven at 95 °C for 6 h.	1.00 wt%	N/A	3.46–3.10 eV, (250–600 nm)	N/A	10 W UV lamp	pH: 6.79, dye vol.: 100 mL, dye conc.,: 10.0 mg L^−1^	180	60.0	[65]
Methylene blue	Ni/ZnO	0.2 g/L	Precipitation method	(Ni(NO_3_)_2_·6H_2_O), (Zn(NO_3_)_2_·6H_2_O), calcining the precursor at 500 °C for 2 h in air.	5.0 mol%	8.12	N/A, (365 nm)	20.0–30.0	300 W Hg lamp	pH: 6.8, dye vol.: 50 mL, dye conc.,: 10.0 mg L^−1^	60	98.0	[61]
Indigo carmine	Ni/ZnO	0.02 g/L	Electrochemical method	(Ni(NO_3_)_2_·6H_2_O), NaHCO_3_, calcined at 500 °C for 2 h.	0.5 wt%	10.0	3.1 eV (340 nm)	78.3	UV light	pH: 10.0, dye vol.: N/A, dye conc.,: N/A	90	84.84	[56]
Methyl orange	Cu/ZnO	0.1 g/L	One-step hydrothermal method	ZnSO_4_, and CuSO_4_ were the precursor, and the mixture underwent a 4 h hydrothermal treatment at 180 °C.	1.0 wt%	N/A	(350–400 nm)	30–150	Sunlight illumination	pH: N/A, dye vol.: 50 mL, dye conc.,: N/A	200	88.0	[37]
Methylene blue	Cu/ZnO	0.025 g/L	Co-precipitation method	(Zn (CH_3_COO)_2_⋅2H_2_O),and (Cu(CH_3_COO)_2_·H_2_O) were taken as precursor, and annealing at 600 °C for 3 h.	5.0 wt%	N/A	3.48 eV (356–364 nm)	19.60–132.02	UV light illumination	N/A	60	72.13	[30]
Methyl orange	Ni/ZnO	0.210 g/L	Sol-gel method	(Ni(NO_3_)_2_·6H_2_O), ((CH_3_COO)_2_Zn·2H_2_O), annealed at 400° for 1 h in a muffle furnace.	2.0 wt%	N/A	3.44–3.16 eV, (350–400 nm)	12.91–8.84 nm	80 W UV lamp	pH: 12.0, dye vol.: 120 mL, dye conc.,: 10.0 mg L^−1^	180	94.47	Present work

### 3.7 Kinetic studies

**Fig. 8:**
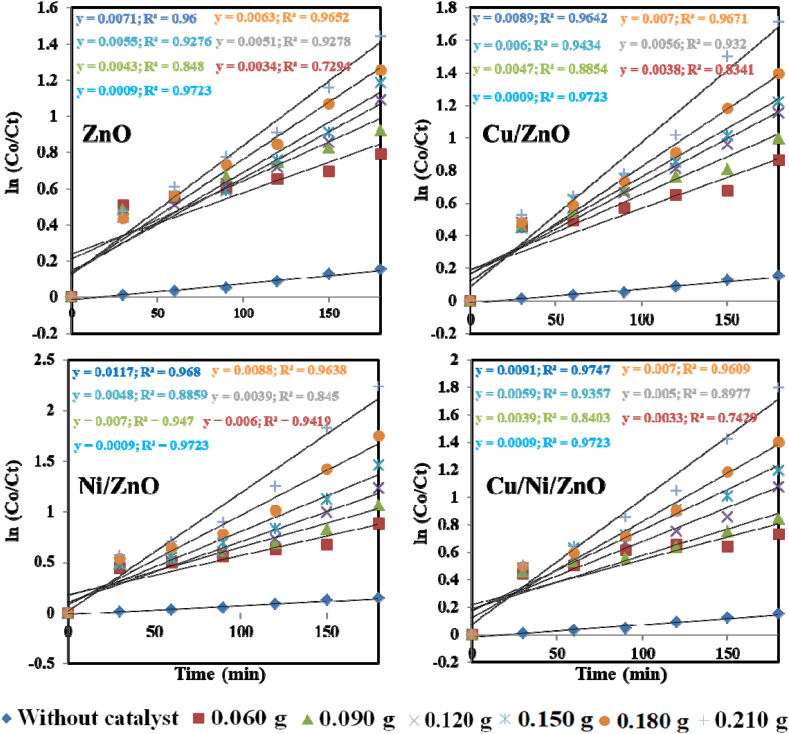
Reaction kinetics of first-order for MO dye removal under the illumination of UV light; (Experimental condition: dose of photocatalyst: 0.060–0.210 g of ZnO, Cu/ZnO, Ni/ZnO, and Cu/Ni/ZnO, pH: 8.7, initial MO dye conc.: 10 mg L^−1^).

The quantitative analysis of the reaction kinetics model of MO dye removal was done by fitting all models including zero-order, first-order, and second-order models to the experimental data. The rate constants with respect to the regression coefficient were measured by plotting ln(Co/Ct) vs. time and the results are shown in Fig. 8. The pure ZnO NPs showed relatively lower photocatalytic activity with k values of 0.0071 min^−1^ after 180 min of UV light illumination, while the k values for other nanocomposites were found to be Cu/ZnO (0.0089 min^−1^), Ni/ZnO (0.0117 min^−1^), and Cu/Ni/ZnO (0.0091 min^−1^), respectively. Additionally, the regression coefficients (R^2^) were observed at 0.96, 0.96, 0.97, and 0.97 for ZnO, Cu/ZnO, Ni/ZnO, and Cu/Ni/ZnO catalysts, respectively, after 180 min illumination of UV light which indicates the first-order reaction was fitted best for MO dye degradation. It was observed that the photodegradation efficiency was increased with the increasing amount of catalyst in the suspension reactor and reached the maximum value of catalyst loading of 0.210 g. This can be explained in terms of the entrance of light into the solution and the availability of active sites on photocatalysts' surfaces [38]. The maximum k value was observed in Ni (2 wt%) doped ZnO nanocomposites due to synergistic effects and available active sites to trap the charge carriers and thus improved the photocatalytic efficiency. The possible reason might be the design of the structure that increased the separation of photogenerated charge carriers and optical absorption in the visible region [28]. It has to be noted that the rate constant of Ni/ZnO composites was almost 1.31 and 1.28 times higher than Cu/ZnO, and Cu/Ni/ZnO nanocomposites respectively. This result revealed the huge enhancement in the degradation rate of ZnO after doping with Ni. Data analysis was used to examine several models in order to study the kinetics of MO dye degradation, and experimental data is shown in Fig. S8–S9.

### 3.8 Photocatalytic reaction mechanism of Ni/ZnO nanocomposite

**Fig. 9:**
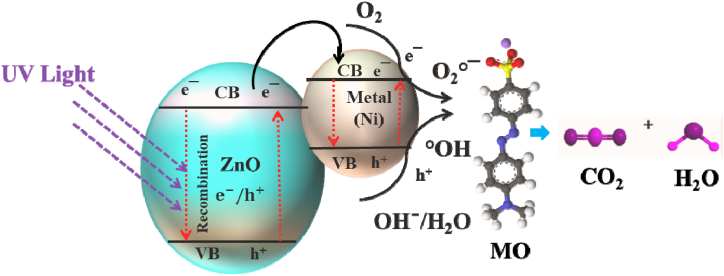
Schematic reaction mechanism of MO dye degradation using Ni/ZnO photocatalys.

The possible photocatalysis mechanism based on dye degradation is illustrated in Fig. 9. The principle of photocatalysis was the adsorption of dye molecules on the catalyst surface while it was illuminated by UV light. An electron (e‾) from the valence band (VB) can be illuminated to the conduction band (CB) with the continuous formation of a hole (h^+^) in the VB when the photon energy was equal to or exceeds the bandgap energy of a photocatalyst semiconductor, [38]. The incorporation of transition metal (Ni) into ZnO created more photogenerated electron-hole traps from the CB/VB of ZnO which fastens the charge carriers and thereby restrained the recombination of photoinduced electrons and holes. The half-filled electronic configuration was enhanced by transferring electrons in the CB of ZnO into Ni dopant. The Ni dopant acts as a trap of electrons and enhanced the lifetime of photogenerated charge carriers, and thereby improved the photocatalytic activity [54,66]. The photogenerated holes can react with water or hydrogen peroxide to produce hydroxyl radical (°OH). Simultaneously, the absorbed oxygen molecules were reduced by the electrons in the CB and forms a superoxide radical anion (O_2_°‾). The generated reactive oxygen species (ROS) were strong powerful oxidants that can oxidize dye and other organics into mineralized products like carbon dioxide (CO_2_), water (H_2_O), and other degradation products [39,66]. The photocatalytic degradation process can be summarized by a series of reactions (Eqs. (5), (6), (7), (8), (9), (10)) which are as follows:

(5) ZnO + hv → ZnO (e_cb_‾ + h_vb_^+^)

(6) ZnO (e_cb_‾ + h_vb_^+^) + O_2_/OH‾/H_2_O → °OH + O_2_°‾

(7) Ni^2+^ + e_cb_‾ (CB) → Ni^+^ (electron trap)

(8) Ni^+^ + O_2_ → Ni^2+^ + O_2_°‾ (electron release)

(9) h_vb_^+^ (VB) + OH‾/H_2_O → °OH

(10) °OH/ O_2_°‾ + MO dye → Degradation products + CO_2_ + H_2_O

In this case, the mainly responsible for the photocatalytic removal of MO dye were the photogenerated electrons and holes. The photodegradation efficiency can be improved by enhancing the charge separation that created defect levels in Ni/ZnO structure. The defect levels inhibited the recombinant photogenerated electron and holes and subsequently increased the photocatalytic effectiveness [67]. Additionally, the photocatalytic activity depended on the bandgap and light utilization capacity of the hybrid Ni/ZnO nanostructure [34].

## 4 Conclusion

A facile sol-gel route was applied to construct metal (Cu or Ni) doped ZnO photocatalysts and characterized using XPS, XRD, SEM with EDX, FTIR, and UV–Vis/DRS spectroscopy analysis. The binding energy with a new chemical state was obtained at 1021.8 eV for ZnO by XPS analysis. XRD and FTIR confirmed the formation of the hexagonal phase with the smaller crystalline size (8.84 ± 0.71 nm) for Ni/ZnO and changes in functional groups were observed. The irregular spherical morphology with decreasing particle diameter (34.43 ± 6.03 to 26.43 ± 4.14 nm) of metal-doped photocatalyst and the elemental composition of nanocomposites were demonstrated from SEM and EDX analyses. UV–Vis/DRS spectra of metal-doped ZnO nanocomposites showed a redshift in the light absorption than pure ZnO NPs. Moreover, the bandgap energy was decreased with the incorporation of metal dopant into ZnO crystal in the range of 3.44 to 3.16 eV. The Ni (2 wt%) doped ZnO nanocomposite exhibited maximum photodegradation efficiency (94.47%) of MO dye (10 mg L^−1^) after 180 min of UV light illumination. The nanocomposite exhibited excellent reusability and found 81.29% efficiency after four consecutive circles. Furthermore, the photodegradation was followed by first-order, and kinetic rate constant values were increased from 0.0009 to 0.117 min^−1^ with the addition of Ni into the ZnO lattice. The improvement of photocatalytic activity was obtained due to the lattice distortion of Ni dopant to suppress recombinant photogenerated electron-hole, porous surface, a red shift in the light absorption, and the synergistic effect of Ni metal on the ZnO surface. Therefore, it can be suggested that the new Ni/ZnO photocatalyst can be applied for the removal of organic dyes from textile effluents.

## Author contribution statement

Md. Rashid Al-Mamun: Conceived and designed the experiments; Performed the experiments; Wrote the paper. Md. Zaveed Iqbal Rokon: Performed the experiments; Wrote the paper. Md. Abdur Rahim; Md. Ikram Hossain: Performed the experiments. Md. Shahinoor IslamORCID; Md. Romzan Ali; Md. Sadek Bacchu: Analyzed and interpreted the data. Hiroki Waizumi; Tadahiro Komeda: Contributed reagents, materials, analysis tools or data. Md. Zaved H Khan: Conceived and designed the experiments; Contributed reagents, materials, analysis tools or data; Wrote the paper.

## Data availability statement

Data will be made available on request.

## Declaration of competing interest

The authors declared that they have no known competing financial interests.

## Declaration of competing interest

The authors declare that they have no known competing financial interests or personal relationships that could have appeared to influence the work reported in this paper.

## Appendix A Supplementary data

Mamun et al. _spl_2023_spl__Revised Manuscript_Heliyon.doc [56-64] Mamun et al. _spl_2023_spl__Revised Manuscript_Heliyon.doc [56-64]

## References

[1] Badi M.Y., Esrafili A., Pasalari H., Kalantary R.R., Ahmadi E., Gholami M., Azari A. (2019). Degradation of dimethyl phthalate using persulfate activated by UV and ferrous ions: optimizing operational parameters mechanism and pathway. J. Environ. Heal. Sci. Eng..

[2] Dodoo-Arhin D., Asiedu T., Agyei-Tuffour B., Nyankson E., Obada D., Mwabora J.M. (2021). Photocatalytic degradation of Rhodamine dyes using zinc oxide nanoparticles. Mater. Today Proc..

[3] Jyothi N.S., Ravichandran K. (2020). Optimum pH for effective dye degradation: Mo, Mn, Co and Cu doped ZnO photocatalysts in thin film form. Ceram. Int..

[4] Aeindartehran L., Ashraf Talesh S.S. (2021). Enhanced photocatalytic degradation of Acid Blue 1 using Ni-Decorated ZnO NPs synthesized by sol-gel method: RSM optimization approach. Ceram. Int..

[5] Hashemi S.Y., Yegane Badi M., Pasalari H., Azari A., Arfaeinia H., Kiani A. (2020). Degradation of Ceftriaxone from aquatic solution using a heterogeneous and reusable O3/UV/Fe3O4@TiO2 systems: operational factors, kinetics and mineralisation. Int. J. Environ. Anal. Chem..

[6] Azari A., Yeganeh M., Gholami M., Salari M. (2021). The superior adsorption capacity of 2,4-Dinitrophenol under ultrasound-assisted magnetic adsorption system: modeling and process optimization by central composite design. J. Hazard Mater..

[7] Bhushan B., Jahan K., Verma V., Murty B.S., Mondal K. (2020). Photodegradation of methylene blue dye by powders of Ni–ZnO floweret consisting of petals of ZnO nanorod around Ni-rich core. Mater. Chem. Phys..

[8] Akter S., Suhan Md B.K., Islam M.S. (2022). Recent advances and perspective of electrocoagulation in the treatment of wastewater: a review. Environ. Nanotechnol. Monit. Manag..

[9] Tariq M., Muhammad M., Khan J., Raziq A., Uddin M.K., Niaz A., Ahmed S.S., Rahim A. (2020). Removal of Rhodamine B dye from aqueous solutions using photo-Fenton processes and novel Ni-Cu@MWCNTs photocatalyst. J. Mol. Liq..

[10] Yasmeen H., Zada A., Liu S. (2019). Dye loaded MnO_2_ and chlorine intercalated g-C_3_N_4_ coupling impart enhanced visible light photoactivities for pollutants degradation. J. Photochem. Photobiol. Chem..

[11] Zada A., Ali N., Subhan F., Anwar N., Ali Shah M.I., Ateeq M., Hussain Z., Zaman K., Khan M. (2019). Suitable energy platform significantly improves charge separation of g-C3N4 for CO2 reduction and pollutant oxidation under visible-light. Prog. Nat. Sci. Mater. Int..

[12] Sahu D. (2020). Degradation of industrial phenolic wastewater using dielectric barrier discharge plasma technique. Russ. J. Appl. Chem..

[13] Abd-Elrahim A.G., Chun D.M. (2021). Room-temperature deposition of ZnO-graphene nanocomposite hybrid photocatalysts for improved visible-light-driven degradation of methylene blue. Ceram. Int..

[14] Zada A., Muhammad P., Ahmad W., Hussain Z., Ali S., Khan M., Khan Q., Maqbool M. (2020). Surface plasmonic-assisted photocatalysis and optoelectronic devices with noble metal nanocrystals: design, synthesis, and applications. Adv. Funct. Mater..

[15] Zada A., Ali N., Ateeq M., Huerta-Flores A.M., Hussain Z., Shaheen S., Ullah M., Ali S., Khan I., Ali W., Shah M.I.A., Khan W. (2020). Surface plasmon resonance excited electron induction greatly extends H2 evolution and pollutant degradation activity of g-C3N4 under visible light irradiation. J. Chin. Chem. Soc. (Taipei, Taiwan).

[16] Amir Zada (2022). Extended visible light driven photocatalytic hydrogen generation by electron induction from g-C3N4 nanosheets to ZnO through the proper heterojunction. Z. Phys. Chem..

[17] Rashid Al-Mamun M., Shofikul Islam M., Rasel Hossain M., Kader S., Shahinoor Islam M., Zaved Hossain Khan M. (2021). A novel and highly efficient Ag and GO co-synthesized ZnO nano photocatalyst for methylene blue dye degradation under UV irradiation. Environ. Nanotechnol. Monit. Manag..

[18] Jenifer A., Sastri M.L.S., Sriram S. (2021). Photocatalytic dye degradation of V2O5 Nanoparticles—an experimental and DFT analysis. Optik.

[19] Sasikala R., Karthikeyan K., Easwaramoorthy D., Bilal I.M., Rani S.K. (2016). Photocatalytic degradation of trypan blue and methyl orange azo dyes by cerium loaded CuO nanoparticles. Environ. Nanotechnol. Monit. Manag..

[20] Al-Mamun M.R., Kader S., Islam M.S. (2021). Solar-TiO_2_ immobilized photocatalytic reactors performance assessment in the degradation of methyl orange dye in aqueous solution. Environ. Nanotechnol. Monit. Manag..

[21] Rashid Al-Mamun M., Hossain K.T., Mondal S., Afroza Khatun M., Shahinoor Islam M., Zaved Hossain Khan D.M. (2022). Synthesis, characterization, and photocatalytic performance of methyl orange in aqueous TiO2 suspension under UV and solar light irradiation. South Afr. J. Chem. Eng..

[22] Verma M., Singh K.P., Kumar A. (2020). Reactive magnetron sputtering based synthesis of WO3 nanoparticles and their use for the photocatalytic degradation of dyes. Solid State Sci..

[23] Subaihi A., Naglah A.M. (2022). Facile synthesis and characterization of Fe2O3 nanoparticles using L-lysine and L-serine for efficient photocatalytic degradation of methylene blue dye. Arab. J. Chem..

[24] Mishra P.K., Biswal S.K., Sahu D. (2022). Synthesis and photocatalytic activity of Ni doped SnO2 nanoparticles for removal of toxic industrial dyes. Mater. Today Proc..

[25] Anbarasu S., Ilangovan S., Usharani K., Prabhavathi A., Suganya M., Balamurugan S., Kayathiri C., Karthika M., Nagarethinam V.S., Balu A.R. (2020). Visible light mediated photocatalytic activity of Ni-doped Al2O3 nanoparticles. Surface. Interfac..

[26] Dawoud T.M.S., Pavitra V., Ahmad P., Syed A., Nagaraju G. (2020). Photocatalytic degradation of an organic dye using Ag doped ZrO2 nanoparticles: milk powder facilitated eco-friendly synthesis. J. King Saud Univ. Sci..

[27] Ahmad I., Aslam M., Jabeen U., Zafar M.N., Malghani M.N.K., Alwadai N., Alshammari F.H., Almuslem A.S., Ullah Z. (2022). ZnO and Ni-doped ZnO photocatalysts: synthesis, characterization and improved visible light driven photocatalytic degradation of methylene blue. Inorg. Chim. Acta..

[28] Ahmad I. (2020). Comparative study of metal (Al, Mg, Ni, Cu and Ag) doped ZnO/g-C3N4 composites: efficient photocatalysts for the degradation of organic pollutants. Sep. Purif. Technol..

[29] Zhao J., Wang L., Yan X., Yang Y., Lei Y., Zhou J., Huang Y., Gu Y., Zhang Y. (2011). Structure and photocatalytic activity of Ni-doped ZnO nanorods. Mater. Res. Bull..

[30] Prasad N., Karthikeyan B. (2017). Cu-doping and annealing effect on the optical properties and enhanced photocatalytic activity of ZnO nanoparticles. Vacuum.

[31] Al-Mamun M.R., Kader S., Islam M.S., Khan M.Z.H. (2019). Photocatalytic activity improvement and application of UV-TiO2 photocatalysis in textile wastewater treatment: a review. J. Environ. Chem. Eng..

[32] Qi K., Liu, Yuan S., Zada A. (2020). Graphitic carbon nitride, a polymer photocatalyst. J. Taiwan Inst. Chem. Eng..

[33] Al-Mamun M.R., Karim M.N., Nitun N.A., Kader S., Islam M.S., Khan M.Z.H. (2021). Photocatalytic performance assessment of GO and Ag co-synthesized TiO_2_ nanocomposite for the removal of methyl orange dye under solar irradiation. Environ. Technol. Innov..

[34] Alam M.W., Aamir M., Farhan M., Albuhulayqah M., Ahmad M.M., Ravikumar C.R., Kumar V.G.D., Murthy H.C.A. (2021). Green synthesis of ni-cu-zn based nanosized metal oxides for photocatalytic and sensor applications. Crystals.

[35] Saleh S.M. (2019). ZnO nanospheres based simple hydrothermal route for photocatalytic degradation of azo dye. Spectrochim. Acta Part A Mol. Biomol. Spectrosc..

[36] Delsouz Khaki M.R., Shafeeyan M.S., Raman A.A.A., Daud W.M.A.W. (2018). Evaluating the efficiency of nano-sized Cu doped TiO_2_/ZnO photocatalyst under visible light irradiation. J. Mol. Liq..

[37] Alatawi N.M., Saad L.B., Soltane L., Moulahi A., Mjejri I., Sediri F. (2021). Enhanced solar photocatalytic performance of Cu-doped nanosized ZnO. Polyhedron.

[38] Türkyılmaz Ş.Ş., Güy N., Özacar M. (2017). Photocatalytic efficiencies of Ni , Mn , Fe and Ag doped ZnO nanostructures synthesized by hydrothermal method: the synergistic/antagonistic effect between ZnO and metals. Journal Photochem. Photobiol. A Chem..

[39] Ben Ameur S., BelHadjltaief H., Duponchel B., Leroy G., Amlouk M., Guermazi H., Guermazi S. (2019). Enhanced photocatalytic activity against crystal violet dye of Co and in doped ZnO thin films grown on PEI flexible substrate under UV and sunlight irradiations. Heliyon.

[40] Karthik K.V., Raghu A.V., Reddy K.R., Ravishankar R., Sangeeta M., Shetti N.P., Reddy C.V. (2022). Green synthesis of Cu-doped ZnO nanoparticles and its application for the photocatalytic degradation of hazardous organic pollutants. Chemosphere.

[41] Shah J., Jan M.R., Khitab F. (2018). Sonophotocatalytic degradation of textile dyes over Cu impregnated ZnO catalyst in aqueous solution. Process Saf. Environ. Protect..

[42] Ahmad M., Ahmed E., Hong Z.L., Jiao X.L., Abbas T., Khalid N.R. (2013). Enhancement in visible light-responsive photocatalytic activity by embedding Cu-doped ZnO nanoparticles on multi-walled carbon nanotubes. Appl. Surf. Sci..

[43] Mittal M., Sharma M., Pandey O.P. (2014). UV-Visible light induced photocatalytic studies of Cu doped ZnO nanoparticles prepared by co-precipitation method. Sol. Energy.

[44] Mishra P.K., Panda N.R., Pati S.P., Biswal S.K., Sahu D. (2021). Studying the effects of Cu doping on structure and photoluminescence properties of SnO 2 nanoparticle with its effectiveness towards the mineralization of toxic industrial dye. ECS J. Solid State Sci. Technol..

[45] Georgekutty R., Seery M.K., Pillai S.C. (2008). A highly efficient Ag-ZnO photocatalyst: synthesis, properties, and mechanism. J. Phys. Chem. C.

[46] Modwi A., Ghanem M.A., Al-Mayouf A.M., Houas A. (2018). Lowering energy band gap and enhancing photocatalytic properties of Cu/ZnO composite decorated by transition metals. J. Mol. Struct..

[47] Chen X., Wu Z., Liu D., Gao Z. (2017). Preparation of ZnO photocatalyst for the efficient and rapid photocatalytic degradation of azo dyes. Nanoscale Res. Lett..

[48] Milenova K., Avramova I., Eliyas A., Blaskov V., Stambolova I., Kassabova N. (2014). Application of activated M/ZnO (M = Mn, Co, Ni, Cu, Ag) in photocatalytic degradation of diazo textile coloring dye. Environ. Sci. Pollut. Res..

[49] Briggs D. (2005). Handbook of Adhesion.

[50] Toloman D., Popa A., Stan M., Stefan M., Vlad G., Ulinici S., Baisan G., Silipas T.D., Macavei S., Leostean C., Pruneanu S., Pogacean F., Suciu R.C., Barbu-Tudoran L., Pana O. (2021). Visible-light-driven photocatalytic degradation of different organic pollutants using Cu doped ZnO-MWCNT nanocomposites. J. Alloys Compd..

[51] Vaiano V., Iervolino G., Rizzo L. (2018). Cu-doped ZnO as efficient photocatalyst for the oxidation of arsenite to arsenate under visible light. Appl. Catal. B Environ..

[52] Beura R., Pachaiappan R., Thangadurai P. (2017). A detailed study on Sn 4 + doped ZnO for enhanced photocatalytic degradation. Appl. Surf. Sci..

[53] Gnanamozhi P., Renganathan V., Chen S.M., Pandiyan V., Antony Arockiaraj M., Alharbi N.S., Kadaikunnan S., Khaled J.M., Alanzi K.F. (2020). Influence of Nickel concentration on the photocatalytic dye degradation (methylene blue and reactive red 120) and antibacterial activity of ZnO nanoparticles. Ceram. Int..

[54] Goswami M. (2020). Enhancement of photocatalytic activity of synthesized Cobalt doped Zinc Oxide nanoparticles under visible light irradiation. Opt. Mater..

[55] Hossain R., Nekouei R.K., Al Mahmood A., Sahajwalla V. (2022). Value-added fabrication of NiO-doped CuO nanoflakes from waste flexible printed circuit board for advanced photocatalytic application. Sci. Rep..

[56] Uma H.B., Ananda S., Nandaprakash M.B. (2019). High efficient photocatalytic treatment of textile dye and antibacterial activity via electrochemically synthesized Ni-doped ZnO nano photocatalysts. Chem. Data Collect..

[57] Juma A.O., Arbab E.A.A., Muiva C.M., Lepodise L.M., Mola G.T. (2017). Synthesis and characterization of CuO-NiO-ZnO mixed metal oxide nanocomposite. J. Alloys Compd..

[58] Dash D., Palai A., Sahu D. (2022). Nanocrystalline gadolinium doped ZnO: an excellent photoluminescent material and efficient photocatalyst towards optoelectronic and environment remedial applications. Ceram. Int..

[59] Zarrabi M., Haghighi M., Alizadeh R. (2018). Sonoprecipitation dispersion of ZnO nanoparticles over graphene oxide used in photocatalytic degradation of methylene blue in aqueous solution: influence of irradiation time and power. Ultrason. Sonochem..

[60] Shanthi S.I., Poovaragan S., Arularasu M.V., Nithya S., Sundaram R., Magdalane C.M., Kaviyarasu K., Maaza M. (2018). Optical, magnetic and photocatalytic activity studies of Li, Mg and Sr doped and undoped zinc oxide nanoparticles. J. Nanosci. Nanotechnol..

[61] Mohd Omar F., Abdul Aziz H., Stoll S. (2014). Aggregation and disaggregation of ZnO nanoparticles: influence of pH and adsorption of Suwannee River humic acid. Sci. Total Environ..

[62] Yang C., Yu J., Li Q., Yu Y. (2017). Facile synthesis of monodisperse porous ZnO nanospheres for organic pollutant degradation under simulated sunlight irradiation: the effect of operational parameters. Mater. Res. Bull..

[63] Yu X., Meng D., Liu C., Xu K., Chen J., Lu C., Wang Y. (2014). Enhanced photocatalytic activity of Fe-doped ZnO nanoparticles synthesized via a two-step sol-gel method. J. Mater. Sci. Mater. Electron..

[64] Ba-Abbad M.M., Takriff M.S., Benamor A., Mohammad A.W. (2016). Synthesis and characterisation of Co2+-incorporated ZnO nanoparticles prepared through a sol-gel method. Adv. Powder Technol..

[65] Shah A.A., Bhatti M.A., Tahira A., Chandio A.D., Channa I.A., Sahito A.G., Chalangar E., Willander M., Nur O., Ibupoto Z.H. (2020). Facile synthesis of copper doped ZnO nanorods for the efficient photo degradation of methylene blue and methyl orange. Ceram. Int..

[66] Thein M.T., Pung S.Y., Aziz A., Itoh M. (2016). Effect of Ni coupling on the photoluminescence property and photocatalytic activity of ZnO nanorods. J. Taiwan Inst. Chem. Eng..

[67] Sahu K., Kuriakose S., Singh J., Satpati B., Mohapatra S. (2018). Facile synthesis of ZnO nanoplates and nanoparticle aggregates for highly efficient photocatalytic degradation of organic dyes. J. Phys. Chem. Solid..

