# Supplementary material for: Enhanced photocatalytic activity of Cu and Ni-doped ZnO nanostructures: A comparative study of methyl orange dye degradation in aqueous solution
Source: Heliyon. 2023 May 22;9(6):e16506. doi: 10.1016/j.heliyon.2023.e16506 (PMC10360600; doi:10.1016/j.heliyon.2023.e16506)
Supplement: Mamun et al. _spl_2023_spl__Revised Manuscript_Heliyon.doc [56-64] [file mmc1.doc]

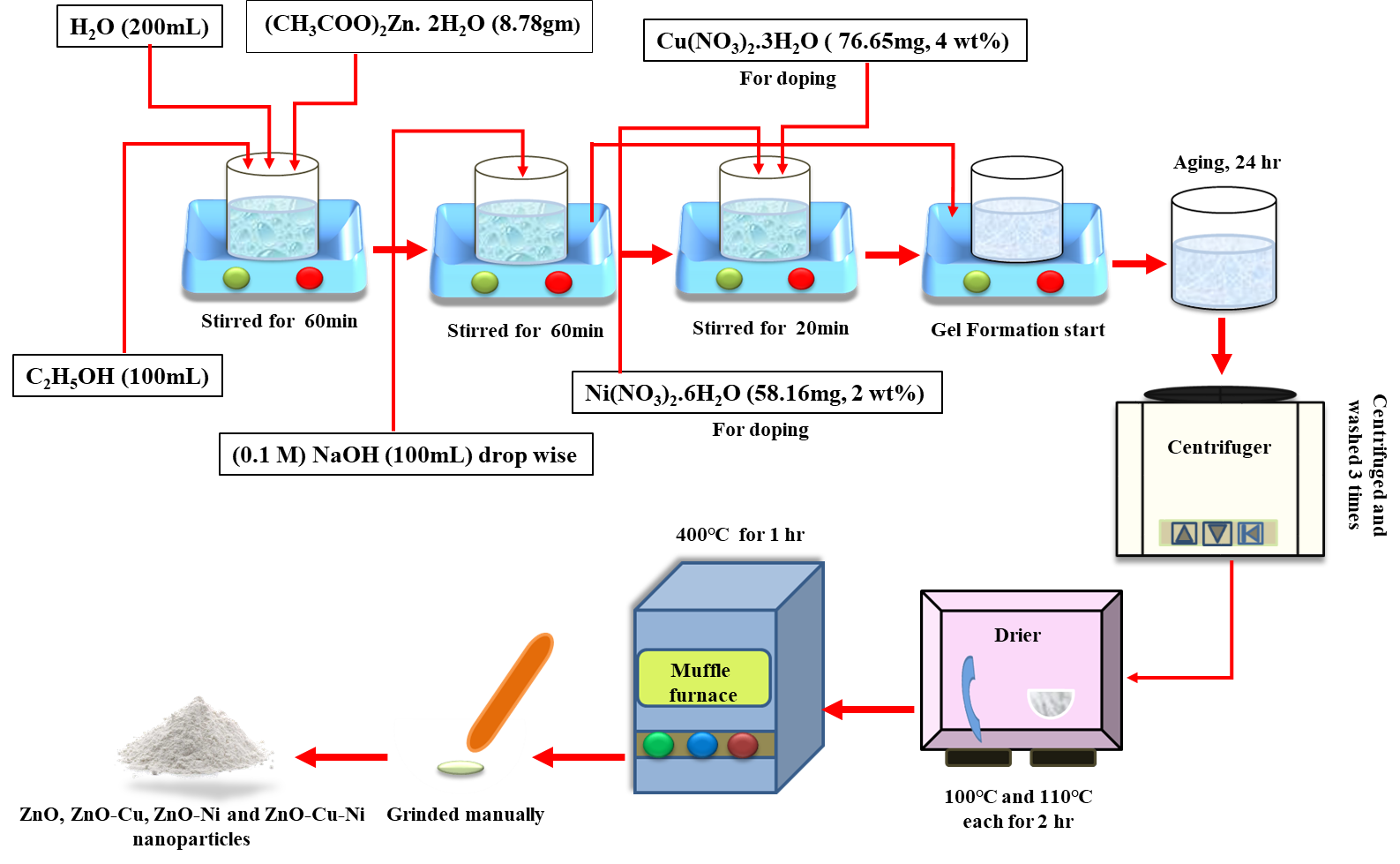


Fig. S1. The schematic diagram of synthesized ZnO based nanocomposites

**
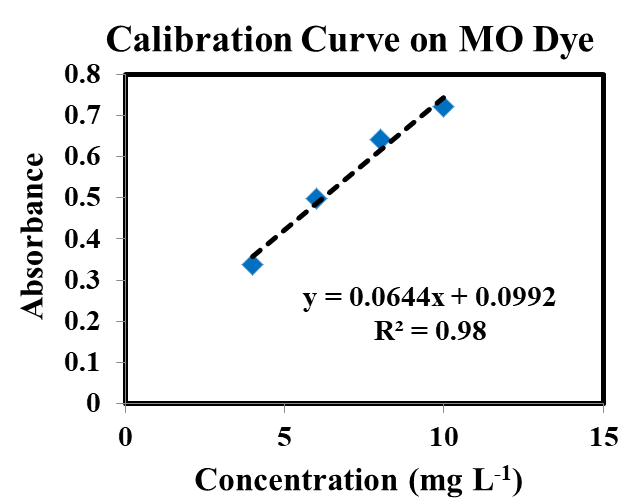
**

Fig. S2. Calibration curve on MO dye


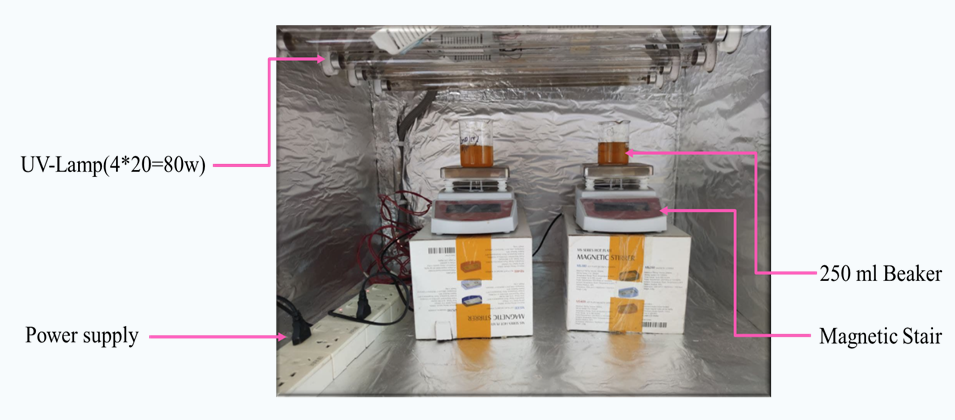


Fig. S3. The experimental setup of photocatalytic reactor

**
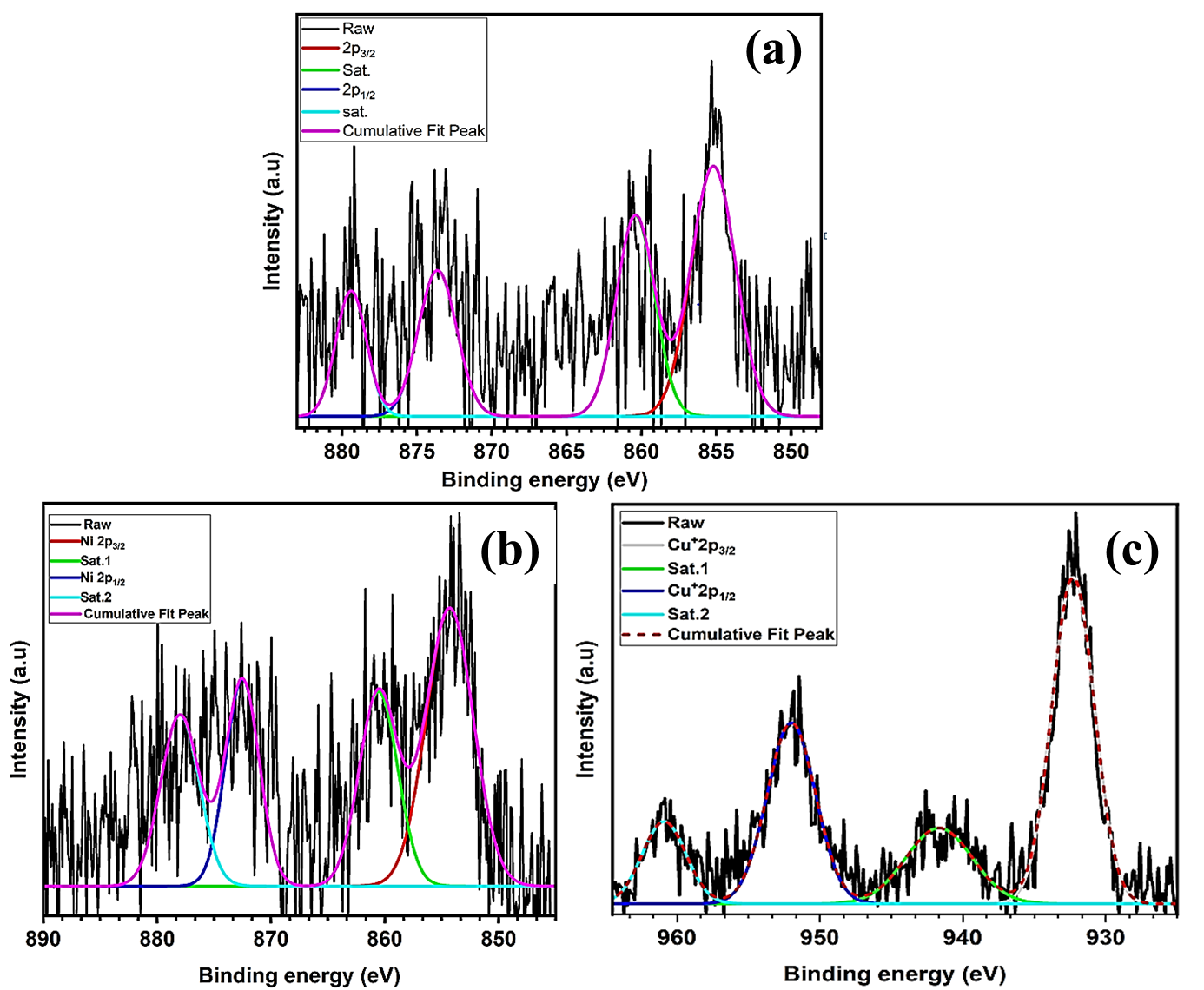
**

Fig. S4. Deconvoluated XPS spectra for Ni and Cu 2p in (a) ZnO_98%_/Ni_2%_, (b) ZnO_94%_/Cu_4%_/Ni_2%_, and (c) ZnO_96%_+Cu_4%_

**
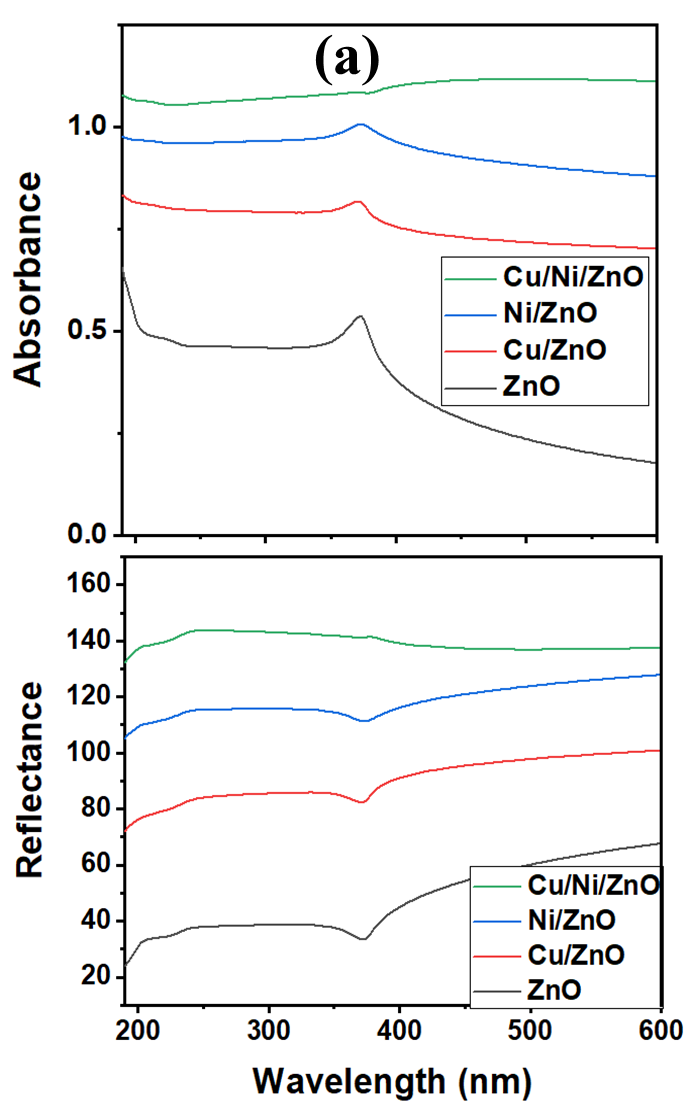
**

Fig. S5. Diffuse reflectance spectra of UV-Vis; (a) absorbance, and (b) reflectance

**
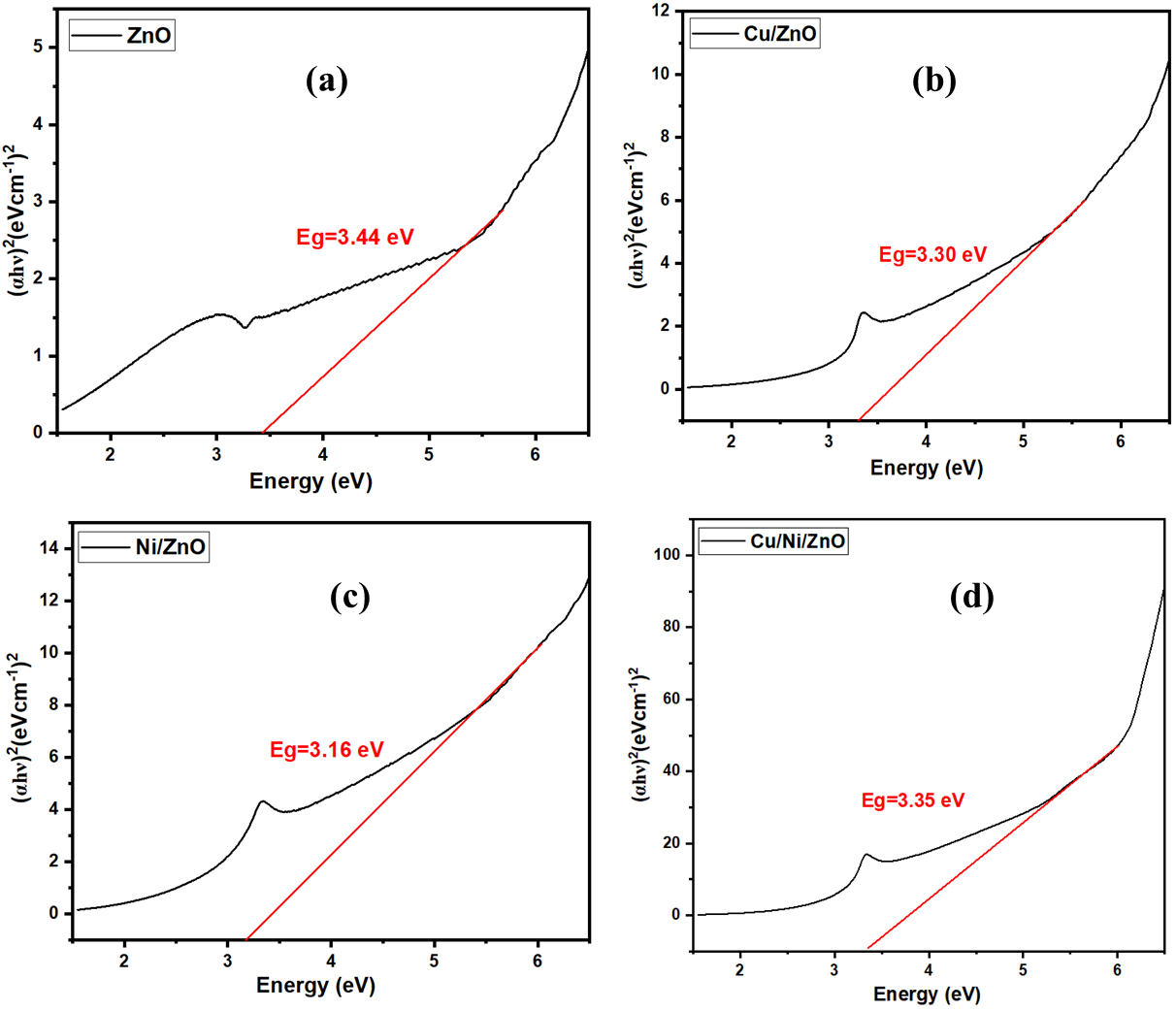
**

Fig. S6. Bandgap energy using Tauc plot; (a) ZnO, (b) Cu/ZnO, (c) Ni/ZnO, and (d) Cu/Ni/ZnO nanocomposite


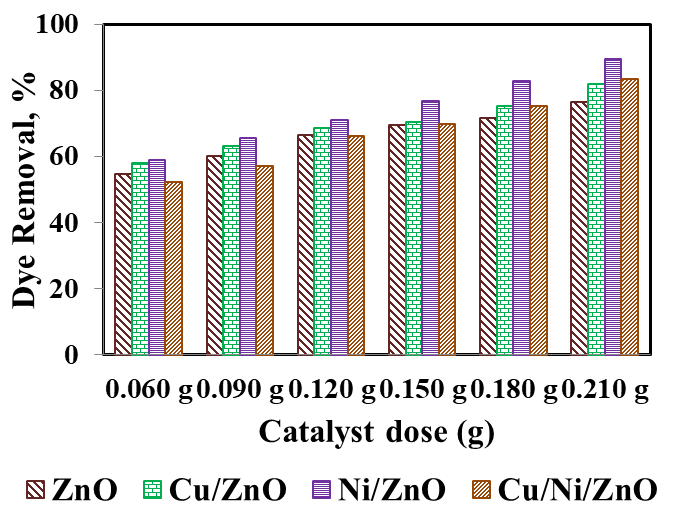


Fig. S7. Comparison of photocatalytic performance of undoped ZnO, Cu/ZnO, Ni/ZnO, and Cu/Ni/ZnO nanocomposites.

**
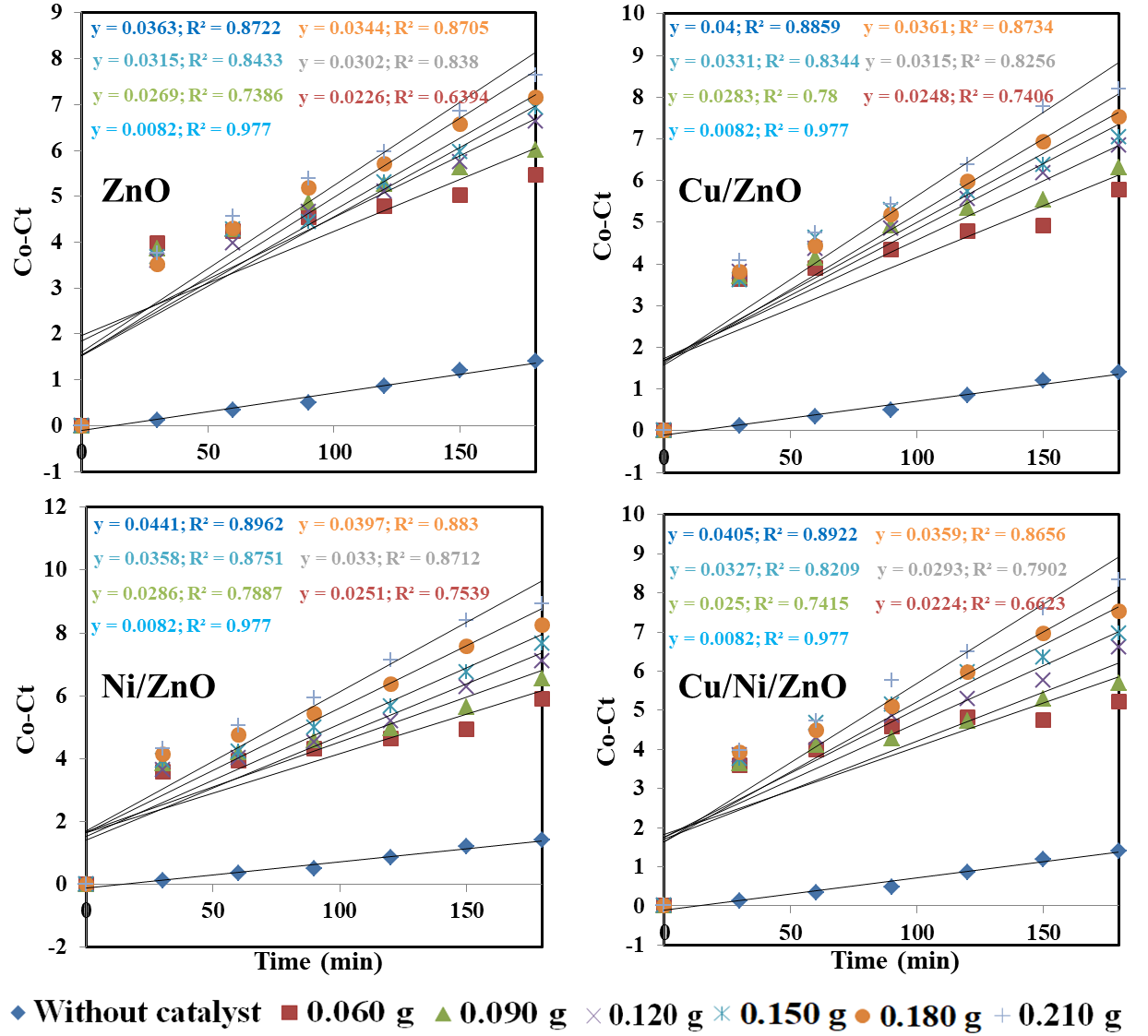
**

Fig. S8. Zero-order reaction kinetics of MO dye removal under the illumination of UV light; (Experimental condition: 0.060-0.210 g dose of ZnO, Cu/ZnO, Ni/ZnO, and Cu/Ni/ZnO; dye conc.: 10 mg L^-1^; pH: 8.7).

**
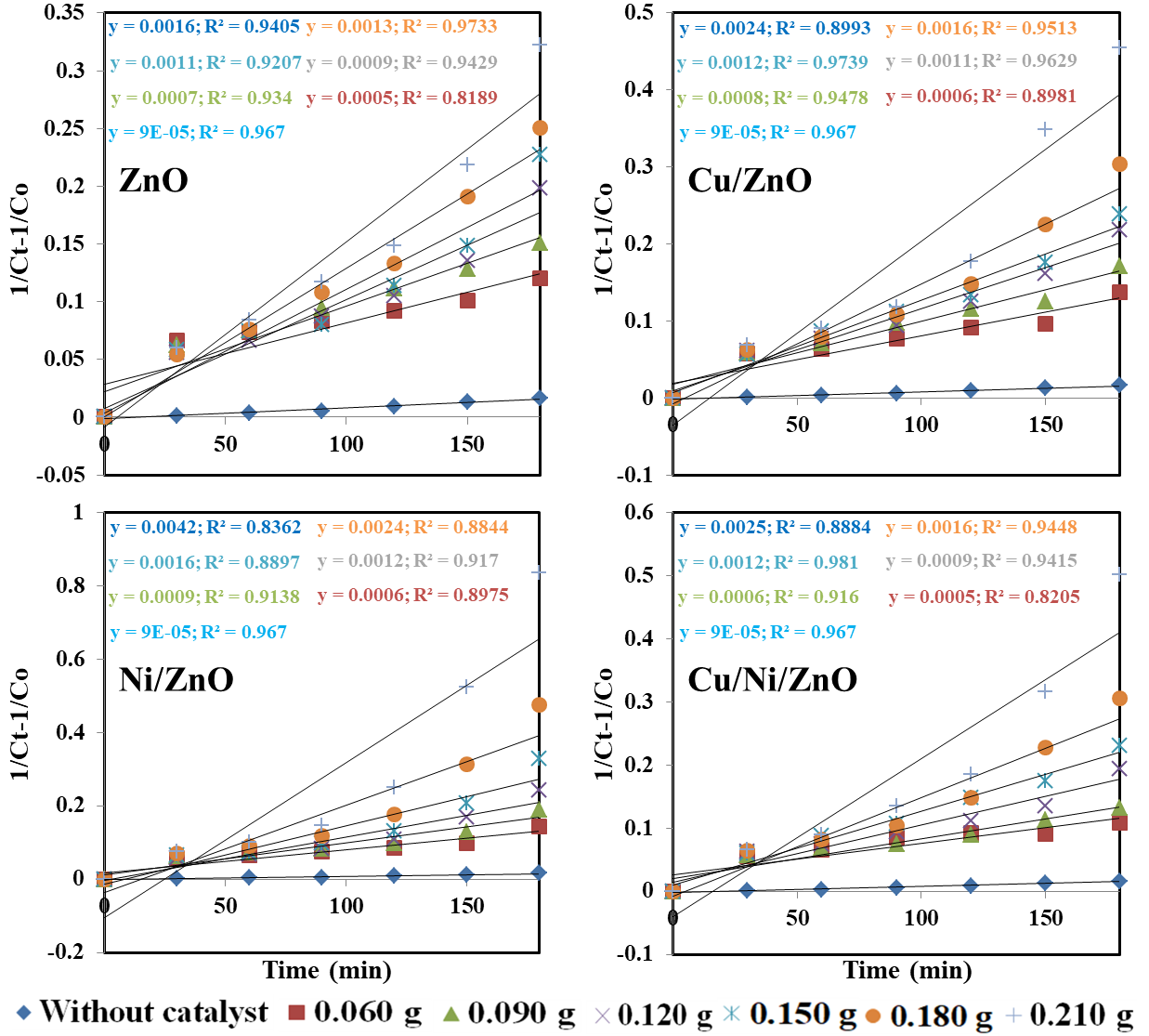
**

Fig. S9. Second-order reaction kinetics of MO dye removal under the illumination of UV light; (Experimental condition: 0.060-0.210 g dose of ZnO, Cu/ZnO, Ni/ZnO, and Cu/Ni/ZnO; dye conc.: 10 mg L^-1^; pH: 8.7).

**Highlights**

- A facile sol-gel method was used to synthesize ZnO based photocatalysts.
- Photocatalysts were characterized by XPS, XRD, SEM with EDX, FTIR, and UV-Vis/DRS analyses.
- Hexagonal structure with crystalline size (17.21-8.84 nm) was observed by XRD analysis
- Bandgap energy of photocatalysts were decreased in the range of 3.44 to 3.16 eV.
- Ni/ZnO exhibited maximum MO dye degradation (94.47%) under UV light illumination.
- Ni/ZnO showed excellent photostabilty and followed first order kinetics.

**Graphical Abstract**

**
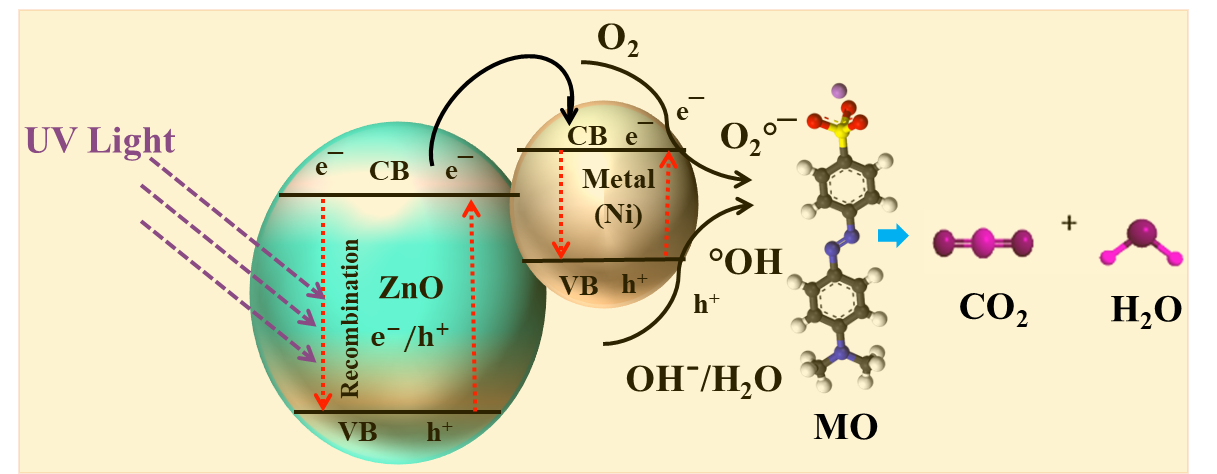
**
